# Targeting PSAT1 to mitigate metastasis in tumors with p53-72Pro variant

**DOI:** 10.1038/s41392-022-01266-7

**Published:** 2023-02-15

**Authors:** Jingwen Jiang, Hai-Ning Chen, Ping Jin, Li Zhou, Liyuan Peng, Zhao Huang, Siyuan Qin, Bowen Li, Hui Ming, Maochao Luo, Na Xie, Wei Gao, Edouard C. Nice, Qiang Yu, Canhua Huang

**Affiliations:** 1 State Key Laboratory of Biotherapy and Cancer Center, West China Hospital, Sichuan University, and Collaborative Innovation Center for Biotherapy, Chengdu, 610041 P.R. China; 2 West China School of Basic Medical Sciences & Forensic Medicine, Sichuan University, Chengdu, 610041 P.R. China; 3 Colorectal Cancer Center, Department of General Surgery, State Key Laboratory of Biotherapy and Cancer Center, West China Hospital, Sichuan University, Chengdu, 610041 P. R. China; 4 Department of Biochemistry and Molecular Biology, Monash University, Clayton, VIC Australia; 5 Cancer Precision Medicine, Genome Institute of Singapore, Agency for Science, Technology, and Research, Biopolis, Singapore, 138672 Singapore

**Keywords:** Cancer therapy, Metastasis

## Abstract

The single-nucleotide polymorphism (SNP) of p53, in particular the codon 72 variants, has recently been implicated as a critical regulator in tumor progression. However, the underlying mechanism remains elusive. Here we found that cancer cells carrying codon 72-Pro variant of p53 showed impaired metastatic potential upon serine supplementation. Proteome-wide mapping of p53-interacting proteins uncovered a specific interaction of the codon 72 proline variant (but not p53^72R^) with phosphoserine aminotransferase 1 (PSAT1). Interestingly, p53^72P^-PSAT1 interaction resulted in dissociation of peroxisome proliferator-activated receptor-γ coactivator 1α (PGC-1α) that otherwise bound to p53^72P^, leading to subsequent nuclear translocation of PGC-1α and activation of oxidative phosphorylation (OXPHOS) and tricarboxylic acid (TCA) cycle. Depletion of PSAT1 restored p53^72P^-PGC-1α interaction and impeded the OXPHOS and TCA function, resulting in mitochondrial dysfunction and metastasis suppression. Notably, pharmacological targeting the PSAT1-p53^72P^ interaction by aminooxyacetic acid (AOA) crippled the growth of liver cancer cells carrying the p53^72P^ variant in both in vitro and patient-derived xenograft models. Moreover, AOA plus regorafenib, an FDA-proved drug for hepatocellular carcinoma and colorectal cancer, achieved a better anti-tumor effect on tumors carrying the p53^72P^ variant. Therefore, our findings identified a gain of function of the p53^72P^ variant on mitochondrial function and provided a promising precision strategy to treat tumors vulnerable to p53^72P^-PSAT1 perturbation.

## Introduction

The complicated and multi-factorial process during pathogenesis and limited therapeutic options leave liver cancer essentially an incurable disease.^1^ Indeed, the development of a therapeutic strategy is challenging due to the genetic and metabolic heterogeneity of liver cancer.^2,3^ Although several molecular classifications have been established, which has deepened our understanding of the heterogeneity of liver cancer,^4^ potential classification-specific therapeutic targets remain to be identified. Therefore, identifying the genetic alterations and subsequent dysregulated signaling pathways will provide a deeper understanding of the subtype classification for liver cancers and hold great potential for identifying novel therapeutic targets. The accumulation of point mutations during evolution, together with random selection, has made single nucleotide polymorphisms (SNPs) the most common event of genetic variation in the human genome.^5^ Indeed, the SNPs of compelling candidate genes have been reported to be associated with tumor progression. For example, the SNP of FOXA2 at its binding sites attenuates the binding of FOXA2 and ERα to their targets thus influencing hepatocellular carcinoma (HCC) development.^6^ However, to date, only a modest number of liver cancer-associated SNPs have been identified, and fewer molecular bases have been revealed. Therefore, further studies focused on the biological function and underlying mechanism of SNPs in tumor progression will shed new light on the classification of liver cancer and provide novel therapeutic targets for precision therapy.

*TP53* is one of the most frequently mutated tumor suppressor genes in cancer.^7^ The somatic mutations of p53 are generally believed to be associated with the inactivation of p53, which results in death evasion of tumor cells and subsequent tumor progression.^8^ However, the roles of germline SNPs found in the coding region of *TP53* in tumorigenesis and tumor development remain largely elusive. A common germline SNP found in *TP53* at its codon 72 (rs1042522) is one of the most widely studied variations of *TP53*, which always presents as Pro 72 (72 P) or Arg 72 (72 R). Although studies based on GWAS have indicated that the codon 72 SNP of p53 is not a risk factor during tumorigenesis, it has been reported that the codon 72 variants display significant effects on the p53-interaction proteins, such as p63 and p73, leading to the distinct outcome of DNA damage and apoptosis in cancer cells.^9,10^ Moreover, the 72 R form of p53 was found to act as a “gain of function (GOF)” variant.^11^ Meanwhile, the codon 72 proline variant of p53 has been revealed to predict primary resistance to chemotherapy,^12,13^ although the underlying mechanisms remain obscure and await further investigation. Considering the important role of p53 in tumorigenesis and tumor development, deciphering the functions of p53 codon 72 variants holds great potential for disease risk stratification and therapeutic decision-making for liver cancer patients.

Cancer cells develop different metabolic adaptation patterns to survive metabolic stress and fulfill the demand of cell proliferation. For example, the Warburg effect describes that cancer cells prefer to utilize aerobic glycolysis instead of oxidative phosphorylation (OXPHOS) to generate ATP and building blocks for rapid proliferation.^14^ Such metabolic adaptations emerge as one of the cancer hallmarks and could be driven by multiple genetic alterations such as *PI3KCA* mutation, *KRAS* mutation, *TP53* loss, and *MYC* amplification.^15^ Recently, metabolic reprogramming associated with active mitochondrial function and OXPHOS has been reported crucially required for several tumor metastases through fueling anabolic metabolism and enhancing ATP generation.^16,17^ Nevertheless, the mechanism underlying the metabolic switch has not yet been fully understood. Previous studies have indicated that p53 could influence a range of metabolic pathways including glycolysis, OXPHOS, and serine synthesis mediated-antioxidant responses. Notably, p53 activation has been reported to be essential for cell survival under glucose starvation or serine deprivation.^18,19^ However, the underlying mechanism of p53 for regulating cell growth under altered extracellular serine or glucose levels, and the connection between the serine synthesis pathway and metabolic switch remain largely unknown. Specifically, it is worth noting that GWAS-based studies indicated that the codon 72 SNP of p53 was closely linked with metabolic diseases such as type-II diabetes and coronary heart disease, suggesting the potential role of p53 codon 72 SNP in regulating metabolic pathways.

In this study, we found that tumor cells with different p53-72 SNP variants have distinct responses to serine supplementation. Interestingly, we found that p53-72Pro showed a specific interaction with phosphoserine aminotransferase 1 (PSAT1), which acted to promote the mitochondrial function and metastatic potential of tumor cells expressing p53-72Pro by facilitating the nuclear translocation of peroxisome proliferator-activated receptor-γ coactivator 1α (PGC-1α). We further demonstrated that aminooxyacetic acid (AOA) could impede the p53-72Pro/PSAT1 interaction and combinational use of AOA and regorafenib, an FDA-proved drug for HCC and colorectal cancer (CRC), showed an obvious synergistic effect on inhibiting tumor growth in patient-derived xenograft models. Our results highlight the non-enzymatic role of PSAT1 in regulating metabolic reprogramming in tumor cells harboring the p53-72Pro variant and provide a potential therapeutic strategy for cancer patients with this variant.

## Results

### PSAT1 is identified as a p53^72P^-interacting protein

**Fig. 1:**
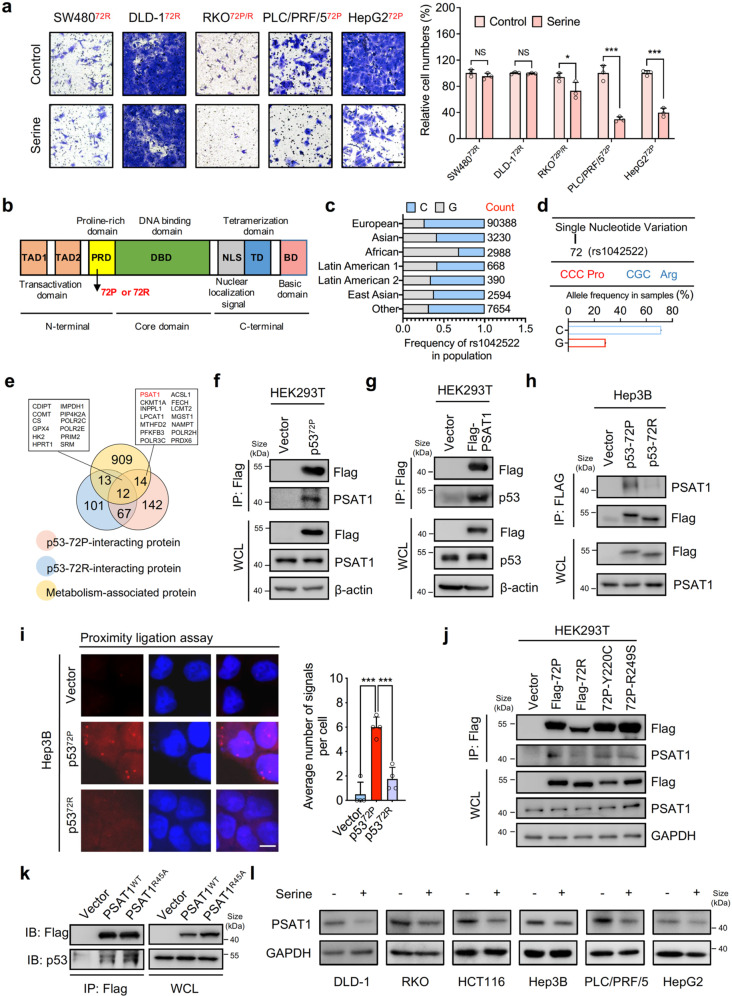
PSAT1 is identified as a p53^72P^-interacting protein. **a** The migration of different tumor cell lines treated with vehicle or serine (500 μM) was detected by transwell assays. Scale bar, 100 μm. Data are means ± s.d. NS not significant, *P < 0.05, ***P < 0.001. **b** The graphic model of functional domains of p53 and the localization of the SNP (rs1042522). **c**, **d** The different variants of rs1042522 in p53. The frequency of SNP rs1042522 in different populations. The data were collected from 105516 samples from NCBI Allele Frequency Aggregator study (https://www.ncbi.nlm.nih.gov/snp/rs1042522). **e** Venn diagram showing the p53-72P-interacting protein, p53-72R-interacting protein, and the overlap of p53-interacting protein with the metabolism-associated protein. **f** Co-immunoprecipitation analysis of the interaction between endogenous PSAT1 and Flag-tagged p53-72P in HEK293T cells. **g** Co-immunoprecipitation analysis of the interaction between Flag-tagged PSAT1 and endogenous p53 in HEK293T cells. **h** Co-immunoprecipitation analysis of the interaction between Flag-tagged p53-72P/72R and endogenous PSAT1 in Hep3B cells. **i** Proximity ligation assay (PLA) in Hep3B cells transfected with vector, p53-72P and p53-72R for the interaction of p53 and PSAT1. Scale bar, 10 μm. Data are means ± s.d. ***P < 0.001. **j** Co-immunoprecipitation analysis of the interaction between Flag-tagged mutant p53 (72P, 72R, Y220C, R249S) and endogenous PSAT1 in HEK293T cells. **k** Co-immunoprecipitation analysis of the interaction between endogenous p53 and Flag-tagged wild type PSAT1 (WT) or mutant PSAT1 (R45A) in HEK293T cells. **l** Immunoblotting analysis of PSAT1 in tumor cell lines treated with or without serine supplementation (500 μM)

To validate the influence of serine supplementation on tumor cell migration, we conducted transwell assay. However, confusing results were observed, as some cell lines exhibited decreased metastatic potential while some kept their metastatic potential (Fig. 1a). When analyzing the genetic background of these tumor cell lines, it was surprising that the SNP (rs1042522) of p53 rather than the mutation of driver genes such as KRAS, MYC or BRAF, had a correlation with the distinct responses to serine supplementation in different tumor cells (Supplementary Fig. S1a). The rs1042522 (codon 72 of p53), a *TP53* SNP located in the proline-rich domain (PRD), has been associated with DNA damage, apoptosis induction, and inflammation in response to stress (Fig. 1b).^20–23^ Indeed, p53^72P^ and p53^72R^ are highly prevalent variants, as shown in the population of a study on 105516 samples (Fig. 1c, d). The codon 72 variants of p53 have been reported to display diverse effects on p53-interacting proteins,^9,10^ we sought to determine if the two major p53 codon 72 variants bind to different proteins to affect the response of tumor cells to serine supplementation. To this end, we used proteomics to compare the p53^72P^ and p53^72R^ interactomes in HEK293T cells (Supplementary Fig. S1b). Overall, 349 proteins were indicated, among which 156 and 114 proteins were identified as individual binding partners for p53^72P^ and p53^72R^, respectively (Fig. 1e). PSAT1 particularly attracted our attention as its critical role in serine biosynthesis.^24^ Moreover, we further confirmed the interaction of endogenous p53 with Flag-PSAT1 using LC-MS analysis (Supplementary Fig. S1c). We then verified the interaction between p53 and PSAT1 in HEK293T cells by exogenous expression of Flag-p53^72P^ or Flag-PSAT1 (Fig. 1f, g). Importantly, as shown in Fig. 1h and Supplementary Fig. S1d, PSAT1 was immunoprecipitated with p53 in cells overexpressing p53^72P^, while this interaction was absent in cells overexpressing p53^72R^. Moreover, the interaction was further confirmed by proximity ligation assay (PLA) in Hep3B cells transfected with vector, p53^72P^ and p53^72R^ plasmids (Fig. 1i). Notably, common hotspot mutations of p53 in HCC cell lines such as Y220C and R249S, had no obvious influence on the interaction of p53^72P^ with PSAT1 (Fig. 1j). Furthermore, we used multiple cancer cell lines containing p53^72P^ or p53^72R^ variants to further validate the endogenous interaction of PSAT1 and the p53^72P^ variant. As shown in Supplementary Fig. S1e–g, the interaction of PSAT1 and p53 can be detected in cells expressing the p53^72P^ variant, such as PLC/PRF/5 and HT29. To rule out the possibility that the enzymatic activity of PSAT1 is required for the interaction between PSAT1 and p53, we constructed a mutant PSAT1 (Flag-PSAT1^R45A^) lacking its glutamate-binding site. As shown in Fig. 1k, mutant PSAT1 can also interact with p53, indicating that the enzymatic activity of PSAT1 might be dispensable for its interaction with p53. To detect if serine supplementation modulates cell migration through regulating the protein expression of PSAT1, we performed immunoblotting assays and found that PSAT1 levels were significantly decreased by serine supplementation (Fig. 1l), indicating that serine supplementation may affect tumor metastasis through modulating PSAT1 level. Taken together, our data reveal that p53^72P^ can specifically interact with PSAT1.

**Fig. 2:**
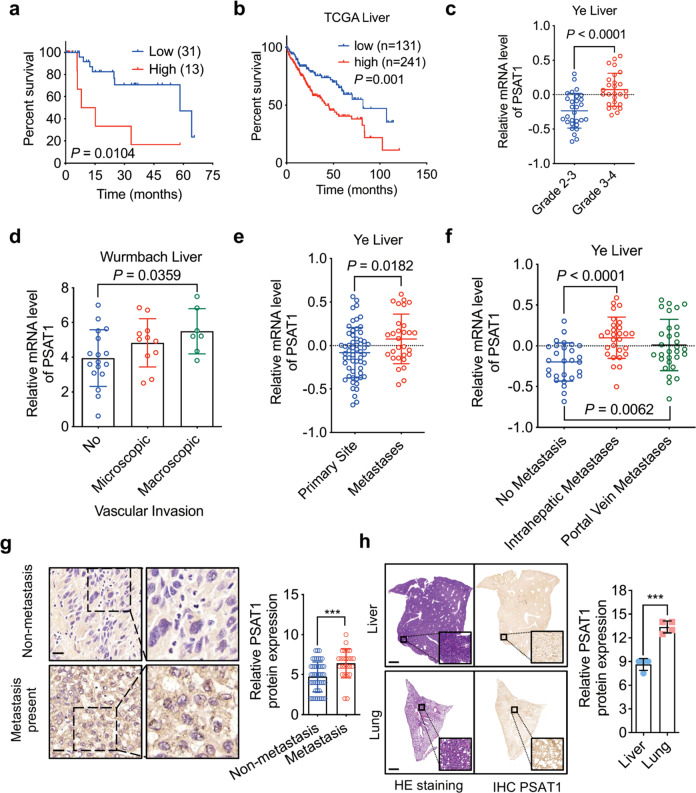
PSAT1 is up-regulated in human metastatic HCC tissues and predicts poor prognosis. **a**, **b** Kaplan-Meier analysis of overall survival rate based on PSAT1 expression in HCC samples and TCGA-Liver dataset. **c** PSAT1 mRNA levels in different grades of HCC patients according to Oncomine data set Ye Liver. **d** PSAT1 mRNA levels in HCC patients with or without vascular invasion according to Oncomine dataset Wurmbach Liver. **e** PSAT1 mRNA levels in primary or metastatic tumor tissues according to Oncomine dataset Ye Liver. **f** PSAT1 mRNA levels in HCC tissues with or without metastases according to Oncomine dataset Ye Liver. **g** IHC analysis of PSAT1 protein level in HCC tissue samples with (*n* = 26) or without (*n* = 46) intrahepatic metastasis. Scale bar, 20 μm. Data are means ± s.d. ***P < 0.001. **h** HE and IHC analysis of PSAT1 protein level in mice liver and lung (intrahepatical mice model). Scale bar, 1000 μm. Data are means ± s.d. ***P < 0.001

To ascertain the role of PSAT1 in liver cancer progression, tissue samples and datasets were analyzed and we found that the protein or mRNA levels of PSAT1 were negatively correlated with the overall survival rate of HCC patients (Fig. 2a, b). In addition, higher PSAT1 levels were found in high-grade tumors and tumors with vascular invasion or other metastases (Fig. 2c–f). Moreover, liver tissues with intrahepatic metastases had relatively higher expression of PSAT1 protein (Fig. 2g, Supplementary Table S1). Importantly, we found that expression of PSAT1 was elevated in lung metastases compared with primary liver cancer (Fig. 2h). Taken together, these data indicate that PSAT1 is up-regulated in human metastatic HCC tissues and predicts poor prognosis.

### PSAT1 depletion impedes the metastatic potential of tumor cells containing p53-72Pro variants

**Fig. 3:**
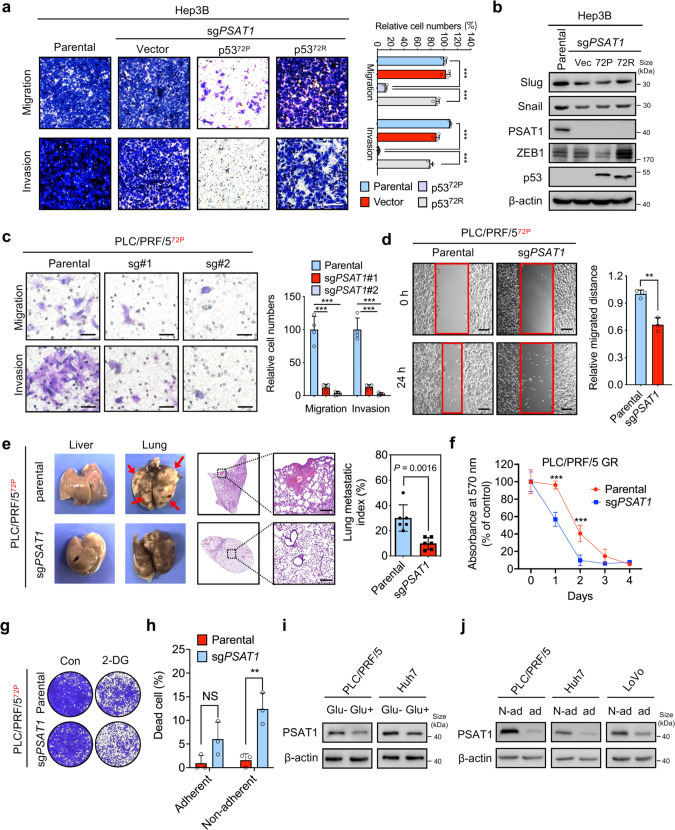
Loss of PSAT1 inhibits the metastatic potential of HCC cells containing p53^72P^. **a** Transwell assay showing the migration and invasion of parental Hep3B cells or PSAT1 knockout Hep3B cells transfected with vector, p53-72P, and p53-72R plasmids. Scale bar, 100 μm. **b** Immunoblotting analysis of indicated proteins in parental or PSAT1 knockout Hep3B cells transfected with vector, p53-72P, and p53-72R plasmids. **c** Transwell assay showing the migration and invasion of parental or PSAT1 knockout PLC/PRF/5 cells. Scale bar, 50 μm. **d** Wound healing assay showing cell migration of parental or PSAT1 knockout PLC/PRF/5 cells after 24 h. Scale bar, 200 μm. **e** Parental or PSAT1 knockout PLC/PRF/5 cells were intrahepatically injected into BALB/c nude mice and the lung metastases were then calculated. Scale bar, 200 μm. **f** Cell viability of parental or PSAT1 knockout PLC/PRF/5 cells treated with glucose restriction for 4 days. **g** Survival fraction of parental or PSAT1 knockout PLC/PRF/5 cells treated with or without 2.5 mM 2-DG. **h** Statistic dead cells of PLC/PRF/5 cells measured by Trypan blue staining. Parental or PSAT1 knockout PLC/PRF/5 cells were treated with or without poly-HEMA for 6 h. **i** Immunoblotting analysis of PSAT1 and β-actin in PLC/PRF/5 and Huh7 cells treated with or without glucose restriction for 24 h. **j** Immunoblotting analysis of PSAT1 and β-actin in indicated cells treated with or without poly-HEMA for 6 h. Data are means ± s.d. NS not significant, **P < 0.01, ***P < 0.001

To elucidate the function of PSAT1 in regulating the metastasis of cells expressing different p53^72^ variants, PSAT1 was knockout in Hep3B cells. Loss of PSAT1 was shown to inhibit the metastasis of Hep3B cells containing the p53^72P^ variant but not in those expressing vector or p53^72R^ variant, indicating that PSAT1 plays an obligatory role in the migration and invasion of cells containing the p53^72P^ variant (Fig. 3a). Given that epithelial-to-mesenchymal transition (EMT) is essential for elevated cell mobility and invasion, we then investigated the effect of PSAT1 knockdown on the expression of EMT markers. PSAT1 knockout or knockdown significantly decreased mesenchymal markers such as ZEB1 in cells expressing p53^72P^ variants (Fig. 3b and Supplementary Fig. S2a, b). Moreover, PSAT1 knockout or knockdown significantly impaired the migration of liver cancer cells (PLC/PRF/5) and CRC cells (HT29) expressing p53^72P^ variants (Fig. 3c, d and Supplementary Fig. S2c), while overexpression of PSAT1 promoted the migration and invasion of HepG2 cells containing p53^72P^ variants (Supplementary Fig. S2d–f). Consistently, PSAT1 knockout could change the morphology of cancer cell lines (PLC/PRF/5) containing p53^72P^ variants (Supplementary Fig. S2g) and increase the mRNA level of *CDH1* (Supplementary Fig. S2h). To further investigate the effect of PSAT1 knockdown on metastatic potential in vivo, PSAT1 knockout PLC/PRF/5 cells were intrahepatically or intravenously injected into BALB/c nude mice, and the lung metastatic index was calculated according to the area and numbers of metastatic nodules. As shown in Fig. 3e and Supplementary Fig. S2i, PSAT1 knockout significantly reduced the lung metastasis of cancer cells in vivo. We next evaluated the effects of PSAT1 knockout on cell survival upon metabolic stresses. As shown in Fig. 3f, g and Supplementary Fig. S2j, k, PSAT1 knockout cells were more vulnerable to glucose restriction or 2-DG treatment. Moreover, PSAT1 knockout increased the proportion of dead cells upon non-adherent treatment (Fig. 3h). In agreement with this, glucose restriction and non-adherent (non-ad) treatment markedly enhanced protein levels of PSAT1 (Fig. 3i, j). On account of this, we speculate that PSAT1 is essential for tumor cells to survive metabolic stresses.

### PSAT1 depletion impedes mitochondrial biogenesis and oxidative phosphorylation in HCC cells containing p53^72P^

**Fig. 4:**
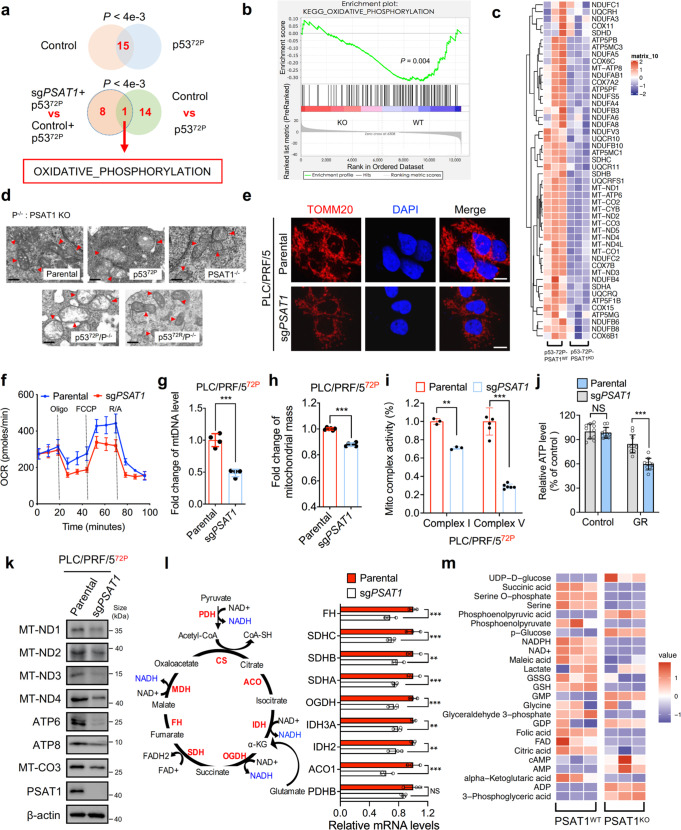
PSAT1 deficiency impedes oxidative phosphorylation in HCC cells containing p53^72P^. **a** Venn diagram showing the overlap of biological signaling pathways in parental or PSAT1 knockout Hep3B cells transfected with vector or p53-72P plasmids. 15 overlapped pathways (*P* < 0.004) were enriched in cells expressing vector and p53-72P, including TCA cycle, valine leucine and isoleucine degradation, type II diabetes mellitus, FC gamma R mediated phagocytosis, oxidative phosphorylation, TGF-β signaling pathway, Parkinsons disease, basal transcription factors, propanoate metabolism, glutathione metabolism, Alzheimers disease, glycolysis gluconeogenesis, drug metabolism cytochrome P450, epithelial cell signaling in helicobacter pylori infection, and cell cycle. **b** GSEA enrichment plot for oxidative phosphorylation in the comparison of Hep3B cells expressing p53-72P/PSAT1^WT^ or p53-72P/PSAT1^KO^. **c** Heatmap for the expression of OXPHOS genes using the fragments per kilobase of transcript per million mapped reads (FPKM) value of RNA-sequence data of Hep3B cells expressing p53-72P/PSAT1^WT^ or p53-72P/PSAT1^KO^ (*n* = 3). **d** Representative TEM image showing the mitochondrial morphology of parental or PSAT1 knockdown Hep3B cells transfected with vector, p53-72P or p53-72R plasmids. Scale bars, 400 nm. **e** Immunofluorescence assays showing the intensity of TOMM20 in parental or PSAT1 knockout PLC/PRF/5 cells. Scale bars, 10 μm. **f** Oxygen consumption rate (OCR) (*n* = 3) analysis using parental or PSAT1 knockout PLC/PRF/5 cells. **g** Mitochondrial DNA (mtDNA) content relative to total cell protein content in PSAT1 knockout PLC/PRF/5 cells normalized to parental PLC/PRF/5 cells. **h** Mitochondrial mass content in PSAT1 knockout PLC/PRF/5 cells normalized to parental PLC/PRF/5 cells. **i** Mitochondrial respiratory chain complex I or V activity detection in parental or PSAT1 knockout PLC/PRF/5 cells. **j** ATP levels of parental or PSAT1 knockout PLC/PRF/5 cells treated with or without glucose restriction (GR). **k** Immunoblotting analysis of indicated proteins in parental or PSAT1 knockout PLC/PRF/5 cells. **l** Transcriptional levels of TCA-associated enzymes based on FPKM value. **m** Heatmaps of TCA metabolites in parental or PSAT1 knockout PLC/PRF/5 cells (*n* = 3). Data are means ± s.d. NS not significant, **P < 0.01, ***P < 0.001

To investigate the role of PSAT1 in cells with p53^72P^ variant, the parental or PSAT1 knockout Hep3B cells were transfected with plasmid expressing vector or p53^72P^ followed by RNA-sequencing. As shown in Fig. 4a–c, PSAT1 knockout prominently restrained the expression of OXPHOS-related mitochondria-encoded genes such as MT-ND1, NDUFB8, and MT-CO3 in cells containing p53^72P^ variants, indicating that PSAT1 may function as a key effector to diverge the metabolic pattern of tumors possessing p53^72P^. To confirm the influence of PSAT1 on mitochondria, transmission electron microscopy (TEM) assay was conducted and the results suggested that HCC cells harboring the p53^72P^ variant, but not p53^72R^, exhibited swelling of the mitochondria when PSAT1 was knocked out (Fig. 4d). In addition, the mitochondrial marker TOMM20 (Fig. 4e), the oxygen consumption rate (OCR) (Fig. 4f), mtDNA levels (Fig. 4g), mitochondrial mass (Fig. 4h), and the activity of mitochondrial complex I and V (Fig. 4i) were dampened in PSAT1 knockdown cells. Notably, PSAT1 knockdown also significantly decreased the ATP level of PLC/PRF/5 cells upon glucose restriction (Fig. 4j). Furthermore, as shown in Fig. 4k, knockdown of PSAT1 dramatically decreased the protein levels of mitochondria-encoded proteins. These data together demonstrate that PSAT1 is critical in maintaining mitochondrial function in tumor cells containing the p53^72P^ variant. Since the mitochondrial tricarboxylic acid (TCA) cycle is coupled with OXPHOS to fuel cellular ATP and is essential to maintain mitochondrial function,^25^ we next performed metabolomics analysis to examine the effect of PSAT1 on the TCA cycle. As shown in Fig. 4l, PSAT1 knockdown reduced the mRNA level of enzymes involved in catalyzing the TCA cycle. Furthermore, intermediates in the TCA cycle such as α-KG, maleic acid, folic acid, and citric acid were significantly decreased in PSAT1 knockout cells (Fig. 4m). Indeed, we found that loss of PSAT1 could inhibit glutamate and methionine metabolism, two pathways that interplay with the mitochondrial pathways (Supplementary Fig. S3a, b). These data suggest that PSAT1 is a pivotal regulator of mitochondrial function in HCC cells containing the p53^72P^ variant.

### Loss of PSAT1 dampens the nuclear translocation of PGC-1α to inhibit TFAM-mediated mitochondrial biogenesis in HCC cells containing the p53^72P^ variant

**Fig. 5:**
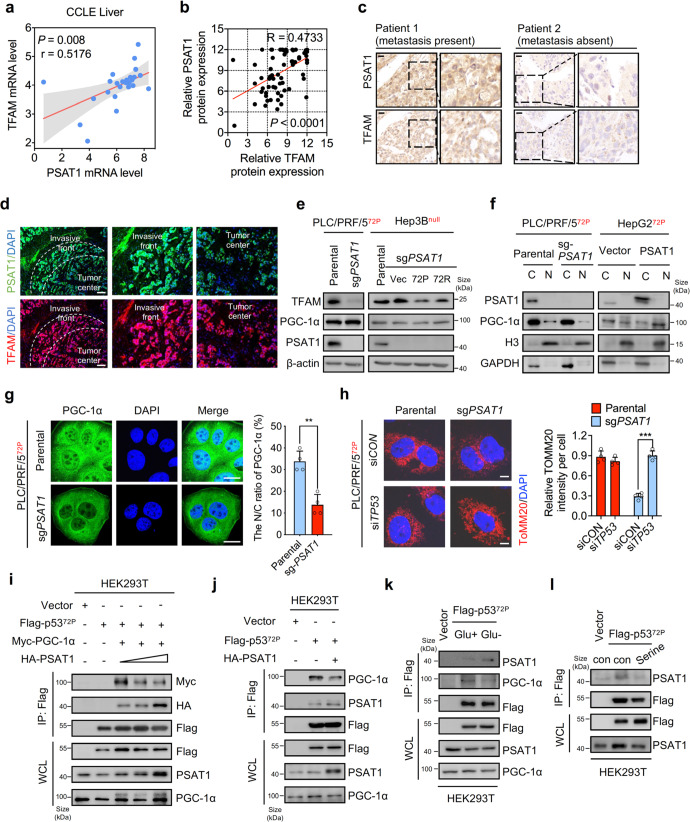
Loss of PSAT1 reduces the nuclear translocation of PGC-1α to inhibit TFAM-mediated mitochondrial biogenesis in HCC cells. **a** CCLE database showing the positive correlation between PSAT1 and TFAM mRNA level in liver cancer cell lines. **b** The Pearson correlation test of the positive correlation between PSAT1 and TFAM protein level in HCC patient samples. **c** Representative immunohistochemical images showing the positive correlation between PSAT1 and TFAM protein levels in human HCC samples. Scale bars, 20 μm. **d** Representative images of tissue immunofluorescence assays showing the PSAT1 and TFAM protein levels in human HCC samples. Scale bars, 100 μm. **e** Immunoblotting analysis of indicated proteins in parental or PSAT1 knockout PLC/PRF/5 cells and Hep3B cells transfected with vector, p53-72P, and p53-72R plasmids. **f** Cell nucleus/cytoplasm fractionation and immunoblotting analysis to show PGC-1α translocation. Histone H3 and GAPDH were used as nuclear and cytoplasmic markers, respectively. PLC/PRF/5 or HepG2 cells were stably knocking out or overexpressing PSAT1. **g** Immunofluorescence assays showing subcellular localization of PGC-1α in parental or PSAT1 knockout PLC/PRF/5 cells. Scale bars, 20 μm. **h** Immunofluorescence assays showing the intensity of TOMM20 after PSAT1 knockout in PLC/PRF/5 cells, in combination with or without si*TP53* treatment. Scale bars, 10 μm. **i** Co-immunoprecipitation analysis of the interaction between extrinsic HA-tagged PSAT1/Myc-tagged PGC-1α and Flag-tagged p53-72P in HEK293T cells. HEK293T cells were transfected with the gradient increasing HA-tagged PSAT1. **j** Co-immunoprecipitation analysis of the interaction between extrinsic HA-tagged PSAT1/endogenous PGC-1α and Flag-tagged p53-72P in HEK293T cells. **k** Co-immunoprecipitation analysis of the interaction between endogenous PSAT1/PGC-1α and Flag-tagged p53-72P in HEK293T cells. HEK293T cells were treated with or without glucose restriction. **l** Co-immunoprecipitation analysis of the interaction between endogenous PSAT1 and Flag-tagged p53-72P in HEK293T cells treated with or without 500 μM serine supplementation. Data are means ± s.d. **P < 0.01, ***P < 0.001

Given that PSAT1 is indispensable for the expression of mitochondria-encoded protein and the maintenance of mitochondrial function, we proposed that PSAT1 may regulate the transcription of the mitochondrial genome. It has been reported that mitochondrial transcription factor A (TFAM) and PGC-1α are important effectors in regulating the expression of mitochondria-encoded proteins.^26^ We therefore analyzed the correlation of expression levels between PSAT1 and TFAM. As shown in Fig. 5a, b, the mRNA or protein expression of PSAT1 was positively associated with TFAM in CCLE liver cancer cell lines and liver cancer patient tissues. In addition, we found that HCC patients with detected metastatic nodes expressed higher PSAT1 and TFAM compared with those cases that had no metastasis (Fig. 5c). Moreover, PSAT1 and TFAM were enriched in the invasive front of liver cancer tissues (Fig. 5d). Consistently, PSAT1 knockout remarkably decreased the protein level of TFAM in cells containing the p53^72P^ variant, but had no noticeable effect on PGC-1α protein level (Fig. 5e). PGC-1α is one of the major transcription factors of TFAM.^27^ Since PSAT1 knockout had no obvious effect on the protein level of PGC-1α, we speculated that PSAT1 might affect the expression of TFAM through regulating the transcriptional activity of PGC-1α and we next examined the nuclear translocation of PGC-1α in PSAT1 knockout cells. Not surprisingly, PSAT1 knockout decreased the nuclear distribution of PGC-1α (Fig. 5f, g), and overexpression of PSAT1 increased its nuclear translocation in cells containing the p53^72P^ variant (Fig. 5f). It is worthwhile noting that PSAT1 knockout had no obvious influence on the protein level of TFAM and the nuclear translocation of PGC-1α in p53-deficient Hep3B cells (Supplementary Fig. S4a–c). Moreover, the sensitivity of p53-deficient Hep3B cells to glucose restriction or 2-DG was not as significant as knocking down PSAT1 in cells containing the p53^72P^ variant (Supplementary Fig. S4d, e and Fig. 3f). Importantly, PSAT1 knockout inhibited the nuclear translocation of PGC-1α only in Hep3B cells expressing the p53^72P^ variant but not in vector or the p53^72R^ variant group (Supplementary Fig. S4f). Together, these data suggest that PSAT1 is critical for the nuclear translocation of PGC-1α and thus promotes the transcription of TFAM in tumor cells expressing the p53^72P^ variant.

It has been reported that p53 can bind to PGC-1α, thus inhibiting its transcriptional activity.^11^ We have verified that PGC-1α interacted with the p53^72P^ variant, which, however, was absent in cells overexpressing the p53^72R^ variant (Supplementary Fig. S4g). This may account for the finding that knocking down PSAT1 only affected PGC-1α translocation in cells overexpressing the p53^72P^ variant. To clarify the role of PSAT1 and p53^72P^ in manipulating the subcellular localization of PGC-1α, si*TP53* was used to detect the nuclear translocation of PGC-1α in parental and PSAT1 knockout cells. As shown in Fig. 5h, si*TP53* treatment could rescue PSAT1 knockout-mediated mitochondrial loss. Moreover, si*TP53* significantly increased the nuclear translocation of PGC-1α in PSAT1 knockout cells (Supplementary Fig. S4h). Consistently, immunofluorescence assays suggested that cells expressing the p53^72R^ variant exhibited higher nuclear distribution of PGC-1α (Supplementary Fig. S4i). Moreover, PGC-1α in cells expressing the p53^72R^ variant was prone to accumulate in the nucleus (Supplementary Fig. S4j).

As we found that PSAT1 could bind specifically to p53^72P^, we then investigated the influence of PSAT1 on the interaction between p53^72P^ and PGC-1α. As shown in Fig. 5i, escalated overexpression of PSAT1 reduced the interaction between p53^72P^ and PGC-1α, indicating that PSAT1 may act as a competitor to restrain the binding of p53^72P^ and PGC-1α. In addition, a Co-IP assay was performed to verify the decreased interaction of p53^72P^ and endogenous PGC-1α in cells transfected with PSAT1 (Fig. 5j). Furthermore, we found that glucose restriction could reduce the interaction of p53 and PGC-1α. As shown in Fig. 5k, glucose restriction markedly decreased the interaction between p53 and PGC-1α, while the binding of p53 and PSAT1 was dramatically enhanced. Moreover, serine supplementation, which can decrease the PSAT1 protein level as described in Fig. 1l, significantly impaired the interaction between PSAT1 and p53-72P (Fig. 5l). In conclusion, PSAT1 may competitively bind to p53^72P^ thus releasing PGC-1α and promoting its nuclear translocation, leading to the maintenance of TFAM-mediated mitochondrial biogenesis.

### AOA impedes metastasis of p53^72P^-containing liver cancer in vivo and in vitro

**Fig. 6:**
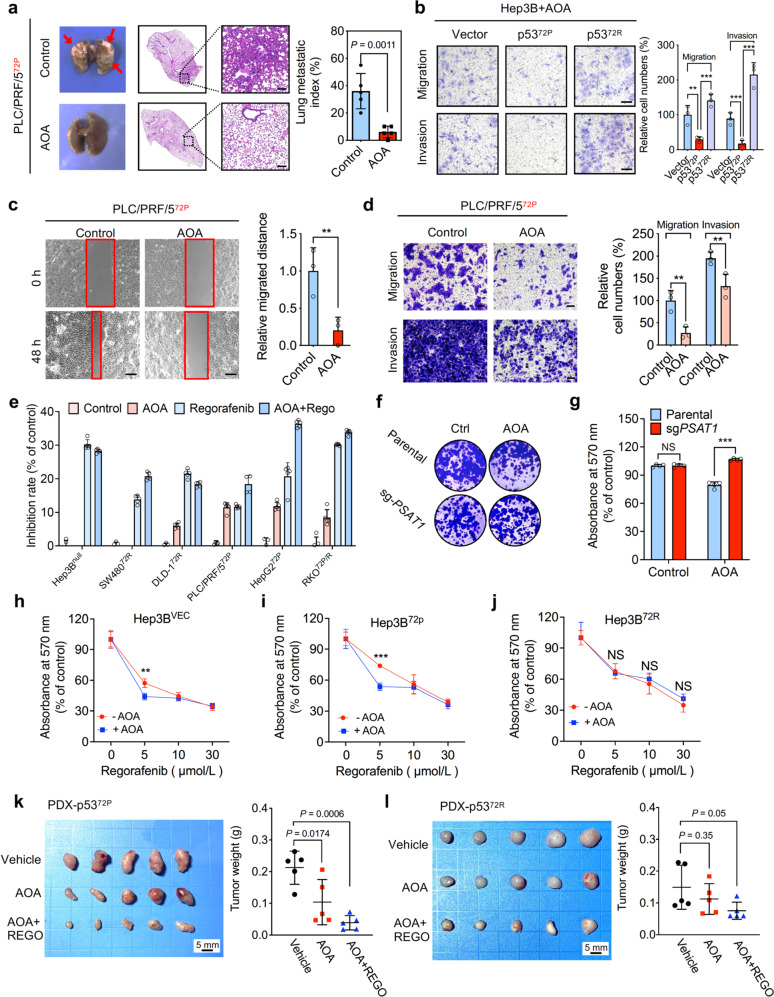
AOA impedes liver cancer growth and metastasis in vivo and in vitro. **a** Parental or PSAT1 knockout PLC/PRF/5 cells were tail intravenously injected into BALB/c nude mice and the mice were treated with vehicle or AOA (10 mg/kg/day). Scale bar, 100 μm. Lung metastases were then calculated after 14-day treatment. **b** Transwell assay showing the migration and invasion of Hep3B cells transfected with vector, p53-72P, and p53-72R, in combination with 20 μM AOA treatment. Scale bar, 100 μm. **c** Wound healing assay showing cell migration of parental or PSAT1 knockout PLC/PRF/5 cells after 48 h, in combination with or without 20 μM AOA treatment. Scale bar, 200 μm. **d** Transwell assay showing the migration and invasion of parental or PSAT1 knockout PLC/PRF/5 cells, in combination with or without 20 μM AOA treatment. Scale bar, 50 μm. **e** The inhibition rate in different cell lines treated with or without 10 μM AOA, in the presence or absence of regorafenib for 24 h. **f** The survival fraction of parental or PSAT1 knockout PLC/PRF/5 cells treated with or without 20 μM AOA. **g** The statistic cell viability of parental or PSAT1 knockout PLC/PRF/5 cells treated with or without 20 μM AOA. **h**–**j** Cell viability of Hep3B cells transfected with vector (**h**), p53-72P (**i**), and p53-72R (**j**) plasmids. Cells were treated with regorafenib alone or AOA (10 μM) plus regorafenib as indicated concentrations. **k** Left: representative images of isolated PDX tumor expressing p53-72P. Xenografts of mice in cohorts were treated with vehicle, AOA (10 mg/kg/day) or AOA plus regorafenib (10 mg/kg/day). Treatment was initiated 24 h after tumors reached 100 mm^3^. Scale bar, 5 mm. Right: the weight of individual tumors in left. **l** Left: representative images of isolated PDX tumor expressing p53-72R. Xenografts of mice in cohorts were treated with vehicle, AOA (10 mg/kg/day) or AOA plus regorafenib (10 mg/kg/day). Treatment was initiated 24 h after tumors reached 100 mm^3^. Scale bar, 5 mm. Right: the weight of individual tumors in left. Data are means ± s.d. NS not significant, **P < 0.01, ***P < 0.001

The above findings highlight a particular metabolic modulating function of PSAT1 by competitively binding to the p53^72P^ variant. Aminooxyacetic acid (AOA) is commonly used as a PSAT1 inhibitor.^28^ We therefore employed AOA for Co-IP analysis and found that the interaction between PSAT1 and p53 was diminished by AOA treatment while the protein level of PSAT1 was not significantly changed (Supplementary Fig. S5a, b). More importantly, AOA treatment significantly increased the interaction of p53 and PGC-1α, indicating that AOA may hold the potential to target the interaction between PSAT1, p53, and PGC-1α to regulate cell metabolism thus interfering with tumor metastasis. As expected, AOA treatment significantly inhibited the lung metastasis of cancer cells expressing the p53^72P^ variant in vivo (Fig. 6a). Moreover, AOA treatment decreased the migration and invasion of HCC cells expressing p53^72P^ variant (Fig. 6b–d). Our previous study showed that the US-FDA-approved drug regorafenib, which is used for the treatment of HCC and CRC, could stabilize PSAT1 by directly binding to it.^24^ Since the interaction of PSAT1 and p53 is critical for the activation of mitochondrial function, we inferred that AOA might enhance the sensitivity of cancer cells to regorafenib. Intriguingly, cells containing the p53^72P^ variant had a lower tolerance to AOA and combinational treatment of AOA and regorafenib (Fig. 6e). Moreover, AOA had no obvious effect on the colony formation capacity of cells when PSAT1 was knocked down, indicating that the anti-tumor effect of AOA may be dependent on PSAT1 (Fig. 6f, g). To further determine the role of AOA in cells containing different p53^72^ variants when exposed to regorafenib, Hep3B cells transfected with vector, p53^72P/R^ and p53^72H/L^ plasmid were treated with AOA. We found that AOA could sensitize tumor cells containing vector and p53^72P/H^ to regorafenib, while exhibiting no obvious synergetic function with regorafenib in cells containing p53^72R/L^ (Fig. 6h–j and Supplementary Fig. S5c). Moreover, rs1042522 was detected and the tissues of 2 patients harboring different p53^72^ variants (Supplementary Fig. S5d, e) were harvested to develop the patient-derived xenograft (PDX) model to evaluate the anti-tumor effect of AOA and combinational use of AOA and regorafenib in vivo. As shown in Fig. 6k, l, PDXs expressing p53^72P^ were more vulnerable to AOA and combinational treatment of AOA and regorafenib, while PDXs expressing p53^72R^ were more resistant. Taken together, these data indicate that AOA holds the potential to inhibit cancer metastasis and improve the anti-tumor effect of regorafenib in patients harboring the p53^72P^ variant.

## Discussion

**Fig. 7:**
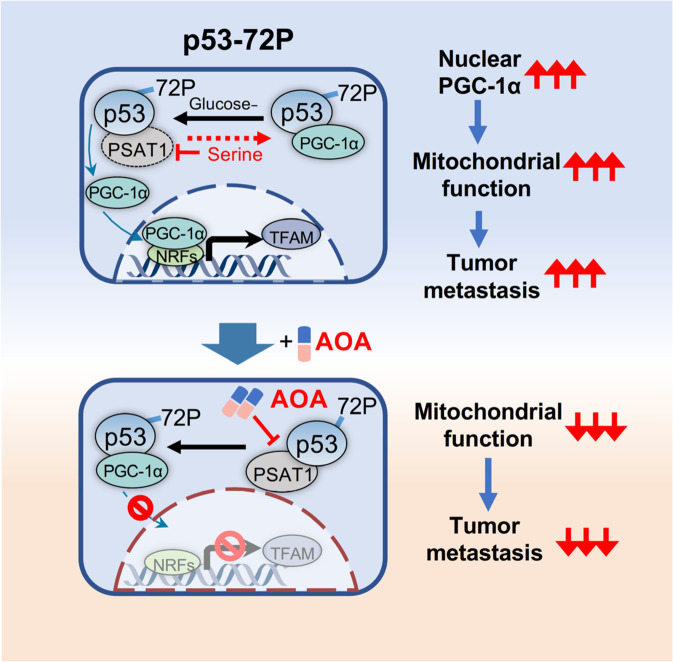
A schematic model illustrating the molecular mechanism of metabolic reprogramming and therapeutic strategies in patients harboring p53^72P^. PSAT1 interacts with p53-72P. Glucose deprivation enhances this interaction and leads to the dissociation of PGC-1α from p53-72P and subsequent nuclear translocation of PGC-1α, which promotes mitochondrial function and tumor metastasis. Interfering with PSAT1 by serine supplementation or AOA treatment can inhibit the interaction of PSAT1 and p53-72P, leading to decreased nuclear translocation of PGC-1α and impaired mitochondrial function and tumor metastasis

Metabolic reprogramming is a hallmark of malignancy^29^ and is closely linked with tumor cell proliferation, metastasis, and drug resistance. It has been widely accepted that the Warburg effect supports the exponential growth and proliferation of cancer cells.^30,31^ In some instances, however, the pathways that encourage the growth of the primary tumor may be distinct from those that promote distant metastasis. Recent studies revealed that enhanced OXPHOS is associated with tumor metastasis, and inhibiting OXPHOS in mouse models reduces metastases in melanoma and breast cancer.^32,33^ This has attracted wide attention that targeting metabolic dependency may serve as a promising strategy for interfering with tumor metastasis and drug resistance. Here, we revealed a moonlighting role of PSAT1 in which PSAT1 interacted with the p53^72P^ variant to regulate mitochondrial function and thus drive cell metastasis, indicating a critical role of PSAT1 in the dynamic switch of metabolism (Fig. 7).

Mounting evidence has confirmed that p53 is involved in a range of cellular metabolic processes, including aerobic glycolysis, OXPHOS, and antioxidant response, besides its pro-apoptotic function during DNA damage.^34^ Typically, the p53 SNP at codon 72 has been implied to determine mitochondrial function in tumor cells, although the underlying mechanism remains unclear.^11^ Here, we screened the discrepant interactome of p53 codon 72 variants and identified PSAT1 as a p53^72P^-interacting protein. The interaction between PSAT1 and p53^72P^ is critical for maintaining the metastatic potential of liver cancer cells through preserving mitochondrial activity. Importantly, this metabolism-regulating function of p53^72P^ seems to be independent of its transcriptional activity. In fact, it has been reported that p53 codon 72 variants display distinct binding ability to their interacting proteins, such as p73.^9^ The ability of p53 to bind to p73 and to neutralize p73-mediated apoptosis was enhanced by the p53^72R^ variant, indicating that the altered affinity of p53 to its interacting protein may be a critical regulatory factor in modulating the function of p53. Indeed, the p53^72P^ variant has been revealed to bind to PGC-1α, which decreased the level of genes transcribed by PGC-1α.^11^ In the present study, we found that PSAT1 was pivotal in the metabolic switch of tumor cells harboring the 72P variant of p53, and the interaction of PSAT1 and p53^72P^ variant was essential for maintaining the activity of PGC-1α. Importantly, several p53 hotspot mutations in HCC, such as Y220C and R249S, had no obvious effect on the interaction of p53 and PSAT1, indicating that PSAT1-mediated metabolic-reprogramming function of p53 may depend mainly on its Pro 72 site. Therefore, targeting the interaction of p53 and PSAT1 might benefit patients with different hotspot mutations, and future pharmacogenomic testing (PGX) may be needed to evaluate this possibility.^35^

Serine is an important metabolite involved in redox homeostasis, nucleotide, and lipid biosynthesis. Previous studies have indicated the critical role of serine in regulating cell proliferation, and inhibition of endogenous serine synthesis through interfering phosphoglycerate dehydrogenase (PHGDH) or PSAT1 may enhance the anti-tumor effect of extrinsic serine restriction.^36,37^ However, complete dietary inhibition of serine seems difficult and the effect of serine on tumor metastasis remains largely unclear. PSAT1 is the second rate-limiting enzyme during serine biosynthesis, which catalyzes the conversion of 3-phosphohydroxypyruvate (PHP) and glutamate to 3-phosphoserine (PS) and α-KG.^38^ The aberrant expression of PSAT1 has been observed in several cancer types including glioblastoma, lung cancer, colorectal cancer, breast cancer, and predicts poor prognosis.^24,39–41^ The mechanism by which the serine biosynthesis-associated activity of PSAT1 facilitates tumor metastasis remains largely obscure and is further confounded by the findings that intervening PSAT1 impedes tumor progression in the presence of exogenous serine,^37^ indicating that PSAT1 may play an oncogenic role beyond its enzymatic function. Indeed, previous research has suggested that PSAT1 induced the protein level of Cyclin D1 through modulating the GSK3β/β-catenin axis, contributing to accelerated cell cycle in breast cancer.^41^ Moreover, it has been indicated that PSAT1 could interact and activate pyruvate kinase M2 (PKM2), an enzyme that controls the final and rate-limiting reaction during the glycolysis pathway, leading to metabolic alterations and subsequent tumor growth.^42^ However, instances of the moonlighting function of PSAT1 in sensing the glucose concentrations and orchestrating metabolic switch are rare. In this study, we found that glucose restriction or non-adherent treatment significantly increased the protein level of PSAT1, indicating that PSAT1 may be involved in the cellular response to metabolic stress. Further study revealed that PSAT1 could competitively interact with p53^72P^ to promote the nuclear translocation of PGC-1α and elicit mitochondrial biogenesis, leading to enhanced OXPHOS and TCA cycle. Indeed, interfering with PSAT1 in cells expressing p53^72P^ significantly impeded tumor cell metastasis, while displaying no obvious effect on cells expressing p53^72R^. Specifically, mutation of the substrate-binding site had little influence on the interaction of PSAT1 and p53^72P^, demonstrating that the enzymatic function of PSAT1 may not be essential. Our finding revealed a non-enzymatic role of PSAT1, in which PSAT1 might be essential for metabolic rerouting during tumor cell metastasis, and targeting PSAT1 may hold the potential to benefit tumor treatment by inhibiting mitochondrial metabolism.

Regorafenib is approved by US-FDA for the treatment of HCC (2017), CRC (2012), and gastrointestinal stromal tumor (2013).^43^ Our previous study confirmed that regorafenib treatment upregulated PSAT1 by directly binding with it and prevented its proteasome-mediated degradation in glioblastoma.^24^ Since upregulated PSAT1 may lead to enhanced mitochondrial function and tumor metastasis in the tumor-possessed p53^72P^ variant, we inferred that the addiction of tumors possessing the p53^72P^ variant to PSAT1 made them more susceptible to PSAT1 inhibitors such as AOA. As expected, AOA significantly strengthened the anti-tumor effect of regorafenib in tumor cells harboring the p53^72P^ variant both in vitro and in vivo. Our study suggested that AOA may be a promising combinational therapeutic option for HCC or CRC patients treated with regorafenib. However, as AOA is a broad-spectrum transaminase inhibitor, a more specific PSAT1 inhibitor may be preferred to assess the potential of targeting PSAT1 in tumors harboring different p53 72 codon variants.

In conclusion, our study underlines the importance of PSAT1 during the progression of tumors with the p53-72P variant. Under glucose restriction, elevated PSAT1 can competitively interact with p53^72P^ to release PGC-1α and promote mitochondrial function. Taken together, we found that PSAT1 may be of particular interest in tumors harboring p53^72P^ to target metabolic reprogramming for preventing tumor progression in clinical practice.

## Materials and methods

### HCC specimens and cell lines

Human HCC specimens were collected from West China Hospital (Chengdu) with informed consent and approval of the Institutional Ethics Committee of Sichuan University.^44^ Liver cancer cell lines Hep3B, PLC/PRF/5, HepG2, Huh7, colorectal cancer cell lines HCT116, HT29, RKO, DLD-1, SW480, LoVo, and HEK293T cells were purchased from American Type Culture Collection or Cell Bank of the Institute of Culture Collection of the Chinese Academy of Sciences. Cells are maintained in Dulbecco’s modified Eagle’s medium (Gibco) supplemented with FBS (10%, BI), penicillin (100 U/mL), and streptomycin (100 μg/mL, Invitrogen) in a 37 °C humidified chamber under 5% CO_2_. To generate PSAT1 stable knockout/knockdown or overexpression cells, indicated cancer cells were infected by lentivirus containing single guide RNA sequence targeting PSAT1, sh*PSAT1* and PSAT1 cDNA-containing plasmids. The plasmids of p53^72^ variants were constructed using Fast Mutagenesis System Kit (TransGen Biotech, cat# FM111-01). The single guided RNAs used in this study are as follows:

Guide RNA#1 forward: 5′-CACCGCCAGGCAGGTGGTCAACTTT-3′; reverse: 5′-AAACAAAGTTGACCACCTGCCTGGC-3′

Guide RNA#2 forward: 5′-CACCGCAGGTGGTCAACTTTGGGCC-3′; reverse: 5′-AAACGGCCCAAAGTTGACCACCTGC-3′.

### Reagents

2-Deoxy-D-glucose (2-DG, a glucose analog that inhibits glycolysis, cat#HY-13966), serine (cat#HY-Y0507), aminooxyacetic acid (AOA, a PSAT1 inhibitor, cat# HY-107994) were purchased from MedChemExpress. Regorafenib (BAY 73-4506, cat#S1178) was purchased from Selleck. DMEM [-] D-glucose was purchased from Gibco (11966-025). Mitochondrial Isolation Kit was purchased from Beyotime Biotechnology (C3601). Mitochondrial DNA Isolation Kit was purchased from Abcam (ab65321). Mitochondrial Respiratory Chain Complex Activity Assaying Kit was purchased from Solarbio (BC0510 and BC1440). ATP Detection Kit was purchased from Beyotime Biotechnology (S0027). Seahorse XF Cell Mito Stress Test Kit was purchased from Seahorse Bioscience (103015-100). Protein A/G beads were purchased from Millipore (Millipore, 16-125, 16-266). Anti-FLAG^®^ M2 Affinity Gel was purchased from Sigma (A2220-5ML).

Primary antibodies are listed below: anti-p53 (Cell Signaling Technology, cat#9282), anti-β-actin (Santa Cruz Biotechnology, cat#sc-69879), anti-PSAT1 (Novus Biologicals, NBP1-32920), anti-FLAG (Cell Signaling Technology, cat#14793S), anti-PGC-1α (Novus Biologicals, NBP1-04676; R&D Systems, MAB10784-SP), anti-HA (Abcam, cat#ab236632), anti-GAPDH (Zen-bio, cat#380626), anti-Slug and anti-ZEB1 (Cell Signaling Technology, cat#49398T), anti-Snail (Cell Signaling Technology, cat#3879), anti-E-Cadherin (Cell Signaling Technology, cat#14472), anti-MT-ND1 (ABclonal, cat#A5250), anti-MT-ND2 (ABclonal, cat#A17968), anti-MT-ND3 (ABclonal, cat#A17969), anti-MT-ND4 (ABclonal, cat#A9941), anti-ATP6 (ABclonal, cat#A17960), anti-ATP8 (ABclonal, cat#A17890), anti-MT-CO3 (ABclonal, cat#A18318), anti-TFAM (Cell Signaling Technology, cat#8076), anti-Histone-H3 (Cell Signaling Technology, cat#4499), anti-Myc-tag (Cell Signaling Technology, cat#2278).

### Animal studies

Male BALB/c nude mice (5-week-old) were purchased from HFK Bioscience Co., Ltd. For the experimental mouse lung metastasis model, 1 × 10^6^ cells were intravenously injected into BALB/c nude mice through the tail vein. For the orthotopic mouse model of liver cancer, 2.5 × 10^5^ cells were intrahepatically injected into the liver of BALB/c nude mice. To test the anti-metastasis effect of AOA, 10 days post-injection of liver cancer cells, mice were randomly grouped and intraperitoneally injected with 0.1 mL vehicle or AOA (10 mg/kg/day) for 14 days. Mice were then euthanized to examine the lung metastasis of tumor cells. Tumor metastasis was monitored by hematoxylin and eosin (HE) staining. All animal studies were performed in accordance with guidelines provided by the Institutional Animal Care and Use Committee of Sichuan University (Chengdu, P.R. China).

### Patient-derived xenograft model

Passage 2 PDX-bearing NSG mice were purchased from Beijing IDMO CO., LTD after validating the status of rs1042522. Tumors were harvested when the tumor reached 800 mm^3^. Tumor tissues were then cut into pieces about 3 mm^3^ and subcutaneously implanted into the NSG mice. When the tumors reached 100 mm^3^, mice were then divided into 3 groups randomly (5 for each group) and intraperitoneally injected with vehicle, AOA (10 mg/kg/day), or AOA (10 mg/kg/day) plus regorafenib (10 mg/kg/day) for 10 days. After drug treatment, mice were euthanized and subcutaneous tumors were harvested for analysis.

### Co-immunoprecipitation (Co-IP) and mass spectrometry (MS) analysis

Cells were harvested and lysed using IP lysis buffer (NaCl 100 mM, EDTA 0.5 mM, Tris-HCl 20 mM, NP-40 0.5%, pH 7.4). Antibodies followed by protein A/G agarose incubation or FLAG-beads were then added and incubated at 4 °C overnight. The beads were then washed 3 times with washing buffer and heated by loading buffer. SDS-PAGE followed by Coomassie brilliant blue G-250 staining was performed to separate the components, followed by excision and dehydration. The dehydrated samples were then reduced by 1,4-dithiothreitol and alkylated by iodoacetamide. Next, peptides were collected after trypsin digestion and purified using ZipTips. The purified samples were then taken for MS analysis. For the detection of metabolites, PLC/PRF/5 cells were harvested and incubated with 80% pre-chilled methanol at −80 °C for 24 h. The samples were then centrifuged at 1,2000 g and the supernatants were collected into 1.5 mL glass bottles and then dried using a Concentrator Plus. The samples were then dissolved by DD water. LC-MS/MS analysis was conducted using an EASY-nLC 1000 nanoflow LC instrument coupled to a Q exactive quadrupole-Orbitrap mass spectrometer.

### Tissue microarray, immunohistochemistry and immunohistofluorescence

A tissue microarray (TMA) comprising 72 liver cancer tissues was used to evaluate PSAT1/TFAM protein expression by immunohistochemistry. The immunohistochemistry assay was performed as described previously.^24^ Briefly, paraffin-embedded slides were rehydrated and then blocked by 3% H_2_O_2_. Next, the slides were incubated in citrate buffer and then underwent antigen retrieval using a microwave. Sections were then blocked with 10% serum and subsequently incubated with indicated primary antibodies. After 3 washes with PBS, for immunohistochemistry assays the sections were treated with MaxVision HRP solution (MXB Biotechnology, 5020) and stained with DAB Peroxidase Substrate (MXB Biotechnology, 0031). For immunohistofluorescence analysis, the slides were incubated with secondary antibody (DyLight 594-conjugated goat anti-mouse IgG, Thermo Fisher Scientific, 35510; DyLight 488-conjugated goat anti-rabbit IgG, Thermo Fisher Scientific, 35552) and then stained with DAPI (Solarbio, C0060). The images were acquired using a DM2500 fluorescence microscope (Leica).

### Transwell assay

For migration assay, 1 × 10^5^ cells were seeded in the upper transwell chamber (Corning, 3422) with serum-free DMEM media. The lower chamber was filled DMEM medium with 10% FBS and cells were incubated for 24 h. For invasion assay, the inserted chamber was pre-coated with matrigel (BD Biosciences, 356234), 2 × 10^5^ cells were planted in the upper chamber and cells were incubated for 48 h. Cells were then fixed with formaldehyde (4%) and stained with crystal violet (0.1%) and the number of migrated cells was calculated using a microscope.

### Immunofluorescence

Cells were seeded onto glass slides (WHB scientific, whb-24-cs) in 24-well culture plates. After indicated treatment, cells were fixed with formaldehyde (4%) and permeabilized with 0.3% Triton X-100. The slides were then washed by PBS and incubated with primary antibodies overnight.^45^ Next, the slides were stained with appropriate secondary antibodies and DAPI. The images were acquired using confocal laser scanning microscopy (Zeiss, LSM780).

### RT-PCR and RNA-sequencing

Trizol (Thermo Fisher Scientific, 15596018) was used to extract total RNA.^46^ Total RNA was then subjected to generate cDNA using PrimeScriptTM RT reagent Kit with gDNA Eraser (Takara, RR047A). RNA-Sequencing was performed with Illumina HiSeq 2000 by Novogene Technology Co., Ltd (Beijing, P.R. China). RT-PCR was performed using Bio-Rad iTaq Universal SYBR Green Supermix (1725271) to identify the mRNA level of target genes.

### Bioinformatic analysis

Raw sequencing reads in the fastq files were mapped to the Gencode (release 34) based on the GRCh37.p13 reference genome and the corresponding GTF file with STAR (version 2.7.5). The table of counts was obtained with FeatureCounts. The voom+limma in the limma package (version 3.42.2) was used for differential expression gene analysis with R (version 3.6.2). Raw library size differences between samples were treated with the weighted trimmed mean method in the edgeR package (version 3.28.1). Read counts were converted to log2-counts-per-million (logCPM) and the mean-variance relationship was modeled with precision weights using voom approach for the differential expression analysis.

### Statistical analysis

Statistical analysis was performed using GraphPad Prism 8.0 to identify the *P*-value. The concordance was examined by Pearson correlation test and the survival curve was determined by log-rank test. The significance between the two groups was calculated by two-tailed Student *t* test or one-way ANOVA. Data are shown as means ± s.d. and the disparity was considered significant if *P* < 0.05. The significance between groups was described as follows: *P < 0.05, **P < 0.01, ***P < 0.001.

## Supplementary information

Supplementary Materials

Figure S1

Figure S2

Figure S3

Figure S4

Figure S5

Table S1

## Data Availability

Analyses of the correlation of PSAT1 level and survival rate of liver cancer patients were performed using data obtained from TCGA liver. Analyses of the expression of PSAT1 in different types of HCC tissues were supported by the Oncomine database (https://www.oncomine.org/). The correlation between PSAT1 and TFAM in cell lines was supported by CCLE database (https://sites.broadinstitute.org/ccle). The pathway analysis was performed with gene set enrichment analysis. The SNP data collected from 105516 samples was supported by NCBI ALFA Allele Frequency (https://www.ncbi.nlm.nih.gov/snp/rs1042522). The RNA-seq data have been deposited in BioProject (https://www.ncbi.nlm.nih.gov/bioproject), accession number PRJNA881488.
