# Supplementary material for: Targeting PSAT1 to mitigate metastasis in tumors with p53-72Pro variant
Source: Signal Transduct Target Ther. 2023 Feb 15;8:65. doi: 10.1038/s41392-022-01266-7 (PMC9929071; doi:10.1038/s41392-022-01266-7)
Supplement: Supplementary file 1 — Supplementary Materials [file 41392_2022_1266_MOESM1_ESM.docx]

Supplementary Materials for

**Targeting PSAT1 to mitigate metastasis in tumors with p53-72Pro variant**

Jingwen Jiang^1,2#^, Hai-Ning Chen^3#^, Ping Jin^1,2#^, Li Zhou^1^, Liyuan Peng^1^, Zhao Huang^1^, Siyuan Qin^1^, Bowen Li^1^, Hui Ming^2^, Maochao Luo^1,2^, Na Xie^2^, Wei Gao^2^, Edouard C. Nice^4^, Qiang Yu^5^, Canhua Huang^1,2*^

Correspondence to: Canhua Huang (hcanhua@hotmail.com). Address: State Key Laboratory of Biotherapy, West China Hospital, and West China School of Basic Medical Sciences & Forensic Medicine, Sichuan University, No. 17, Section 3, South Renmin Rd, Chengdu, P.R. China.

**This PDF file includes:**

Figures. S1 to S5

Table S1


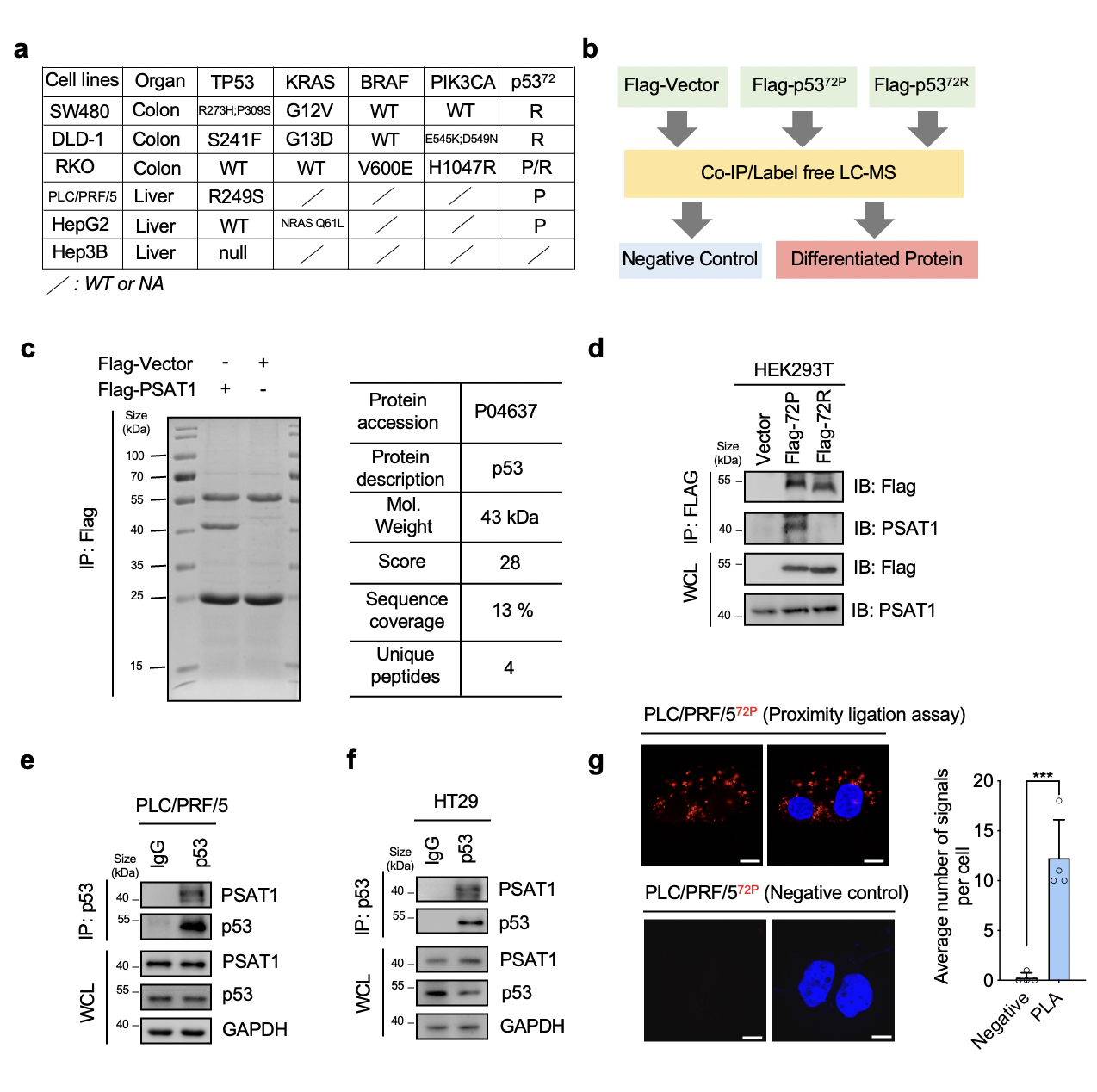


Figure. S1. PSAT1 is identified as a p53^72P^-interacting protein.

**a** A summary for the genetic background information of cell lines used in this study. **b** The graphic workflow for screening p53-72P/72R interaction proteins. **c** Total lysates from HEK293T cells with or without overexpression of Flag-tagged PSAT1 were coimmunoprecipitated with anti-Flag affinity gel agarose, followed by SDS gel electrophoresis and Coomassie brilliant blue staining (left). The table includes mass spectrometric results identifying the PSAT1-binding protein p53 (right). **d** Co-immunoprecipitation analysis of the interaction between Flag-tagged p53-72P/72R and endogenous PSAT1 in HEK293T cells. **e, f** Co-immunoprecipitation analysis of the interaction between endogenous PSAT1 and p53 in PLC/PRF/5 (e) and HT29 cells (f)**. g** Proximity ligation assay in PLC/PRF/5 cells for detecting the interaction of p53 and PSAT1. Scale bar, 10 μm. Data are means ± s.d. ****P* < 0.001.


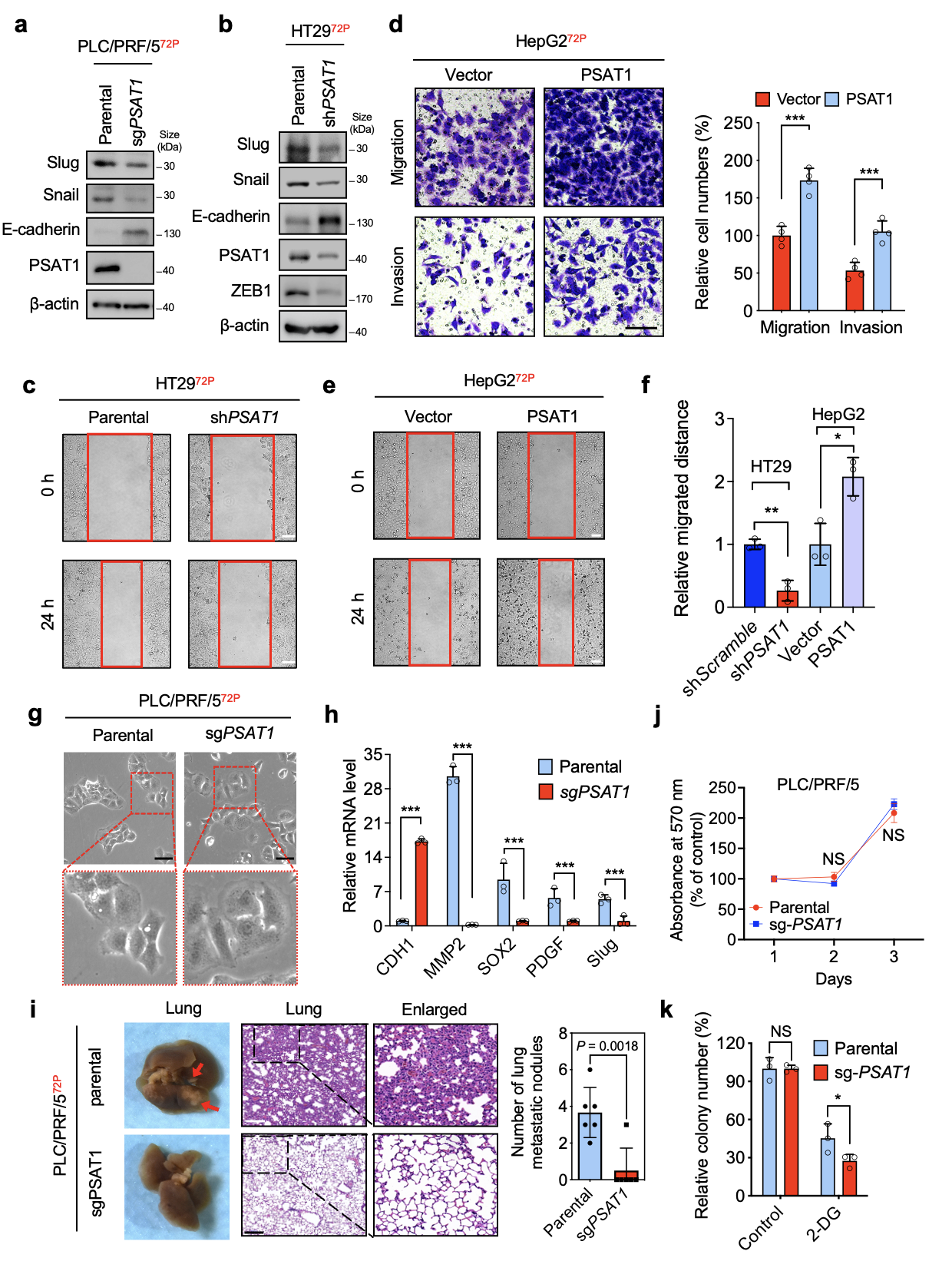


Figure. S2. PSAT1 is essential for the metastatic potential of HCC cells containing p53^72P^.

**a** Immunoblotting analysis of indicated proteins in parental or PSAT1 knockout PLC/PRF/5 cells. **b** Immunoblotting analysis of indicated proteins in parental or PSAT1 knockdown HT29 cells. **c** Wound healing assay showing cell migration of parental or PSAT1 knockdown HT29 cells at indicated times. Scale bar, 100 μm. **d** Transwell assay showing the invasion and migration of HepG2 cells stably transfected with vector or PSAT1. Scale bar, 100 μm. **e** Wound healing assay showing cell migration of HepG2 cells stably transfected with vector or PSAT1 plasmids at indicated times. Scale bar, 100 μm. **f** Related migrated distance of c (left) and e (right). **g** Representative morphology image of parental or PSAT1 knockout PLC/PRF/5 cells. Scale bar, 50 μm. **h** qPCR analysis of the mRNA levels of EMT-associated genes in parental or PSAT1 knockout PLC/PRF/5 cells. **i** Parental or PSAT1 knockout PLC/PRF/5 cells were tail intravenously injected into BALB/c nude mice and lung metastases were then calculated 14 days later. Scale bar, 100 μm. **j** Cell viability of parental or PSAT1 knockout PLC/PRF/5 cells at the indicated time. **k** Relative colony numbers of parental or PSAT1 knockout PLC/PRF/5 cells treated with or without 2.5 mM 2-DG (Fig. 3g). Data are means ± s.d. NS, not significant, **P* < 0.05, ***P* < 0.01, ****P* < 0.001.


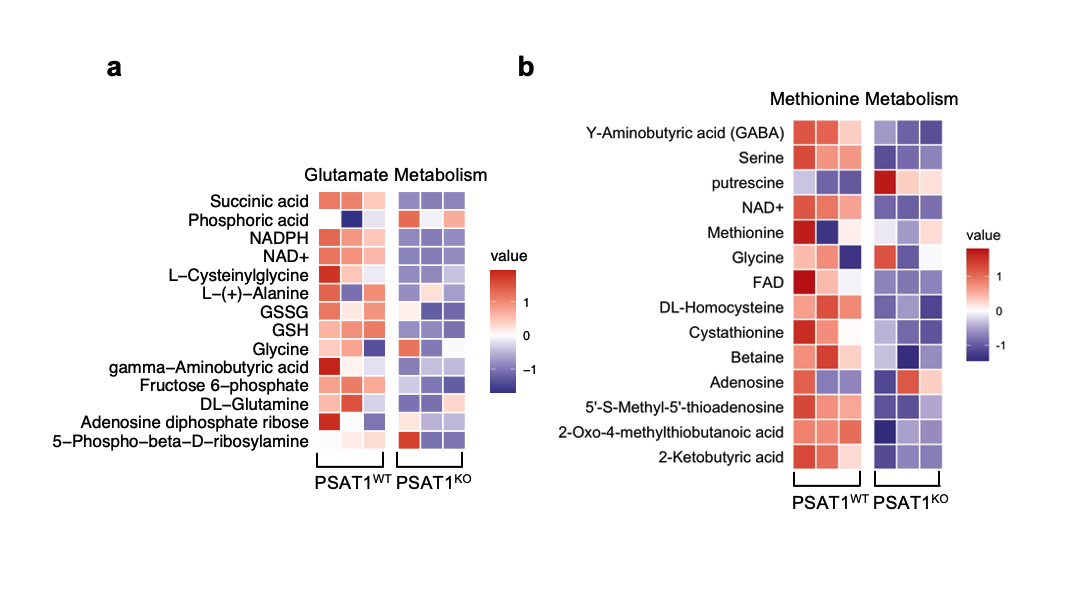


Figure. S3. Loss of PSAT1 impedes mitochondria-related metabolic pathways in HCC cells.

**a** Heatmaps of glutamate metabolism-associated metabolites in parental or PSAT1 knockout PLC/PRF/5 cells (n = 3). **b** Heatmaps of methionine metabolism-associated metabolites in parental or PSAT1 knockout PLC/PRF/5 cells (n = 3).


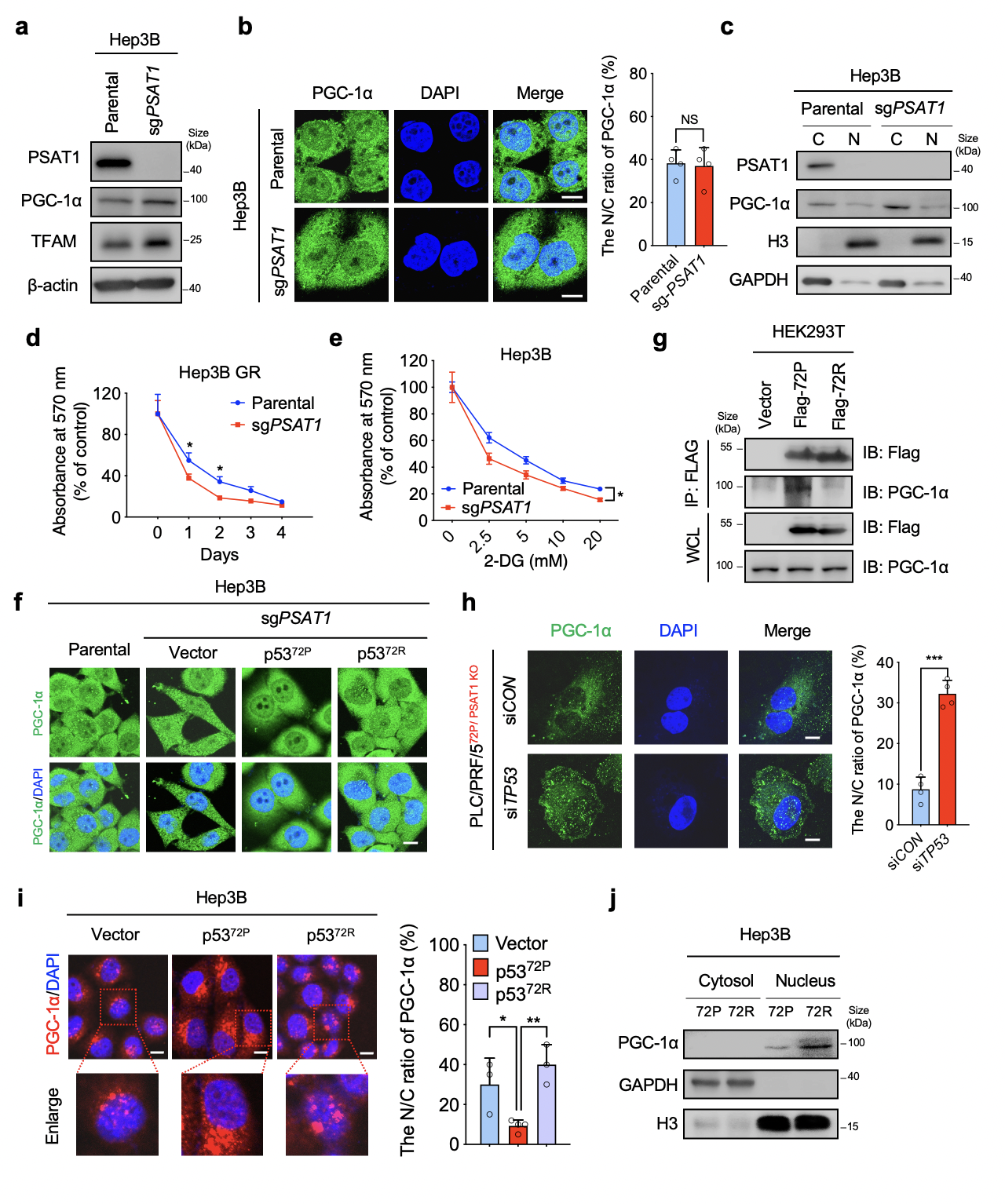


Figure. S4. PSAT1 deletion retards PGC-1α nuclear translocation in cells expressing p53^72P^.

**a** Immunoblotting analysis of indicated proteins in parental or PSAT1 knockout Hep3B cells. **b** Immunofluorescence assays showing subcellular localization of PGC-1α in parental or PSAT1 knockout Hep3B cells. Scale bars, 10 μm. **c** Cell nucleus/cytoplasm fractionation and immunoblotting analysis to show PGC-1α translocation in parental or PSAT1 knockout Hep3B cells. Histone H3 and GAPDH were used as nuclear and cytoplasmic markers, respectively. **d** Cell viability of parental or PSAT1 knockout Hep3B cells. Cells were treated with glucose restriction (GR) for 4 days. **e** Cell viability of parental or PSAT1 knockout Hep3B cells. Cells were treated with 2-DG as indicated concentrations. **f** Immunofluorescence assays showing subcellular localization of PGC-1α in parental or PSAT1 knockout Hep3B cells transfected with vector, p53-72P, p53-72R plasmids. Scale bars, 10 μm. **g** Co-immunoprecipitation analysis of the interaction between endogenous PGC-1α and Flag-tagged p53-72P or p53-72R in HEK293T cells. **h** Immunofluorescence assays showing subcellular localization of PGC-1α in PSAT1 knockout PLC/PRF/5 cells treated with or without si*TP53*. Scale bars, 10 μm. **i** Immunofluorescence assays showing subcellular localization of PGC-1α in Hep3B cells transfected with vector, p53-72P, p53-72R plasmids. Scale bars, 10 μm. **j** Cell nucleus/cytoplasm fractionation and immunoblotting analysis to show PGC-1α translocation in Hep3B cells transfected with p53-72P, p53-72R. Histone H3 and GAPDH were used as nuclear and cytoplasmic markers, respectively. Data are means ± s.d. NS, not significant, **P* < 0.05, ***P* < 0.01, ****P* < 0.001.


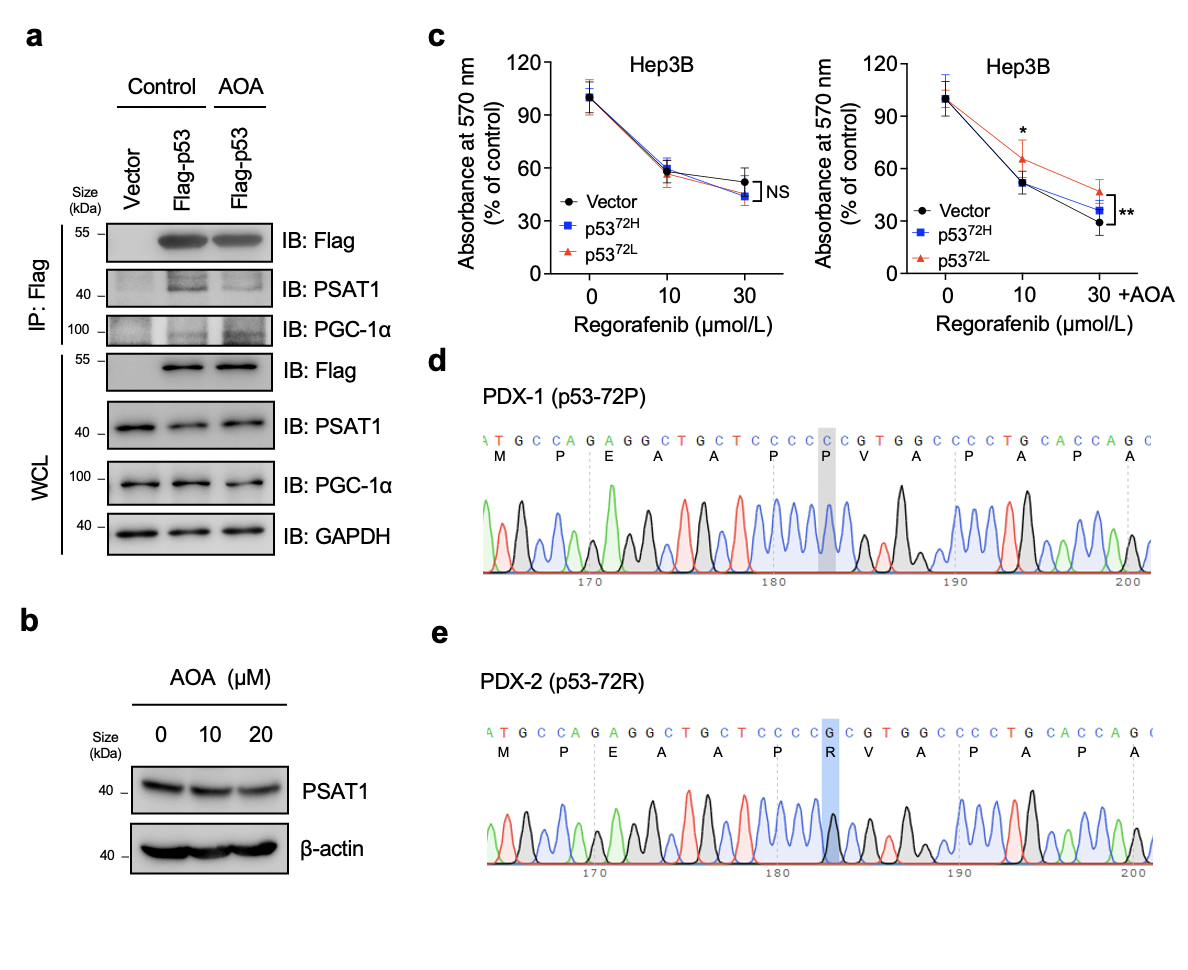


Figure. S5. AOA impedes the interaction between PSAT1 and p53^72P^.

**a** Co-immunoprecipitation analysis of the interaction between endogenous PSAT1/PGC-1α and Flag-tagged p53-72P in HEK293T cells treated with or without 20 μM AOA for 24 hours. **b** Immunoblotting analysis of indicated proteins in PLC/PRF/5 cells treated with or without AOA for 24 hours. **c** Left: cell viability of Hep3B cells transfected with vector, p53-72H and p53-72L plasmids after treatment of regorafenib as indicated for 24 h. Right: cell viability of Hep3B cells transfected with vector, p53-72H and p53-72L plasmids after treatment of regorafenib plus 10 μM AOA for 24 h. **d, e** The sequencing data show the 72 aa variants of p53 in two PDX models. Data are means ± s.d. NS, not significant, **P* < 0.05, ***P* < 0.01.


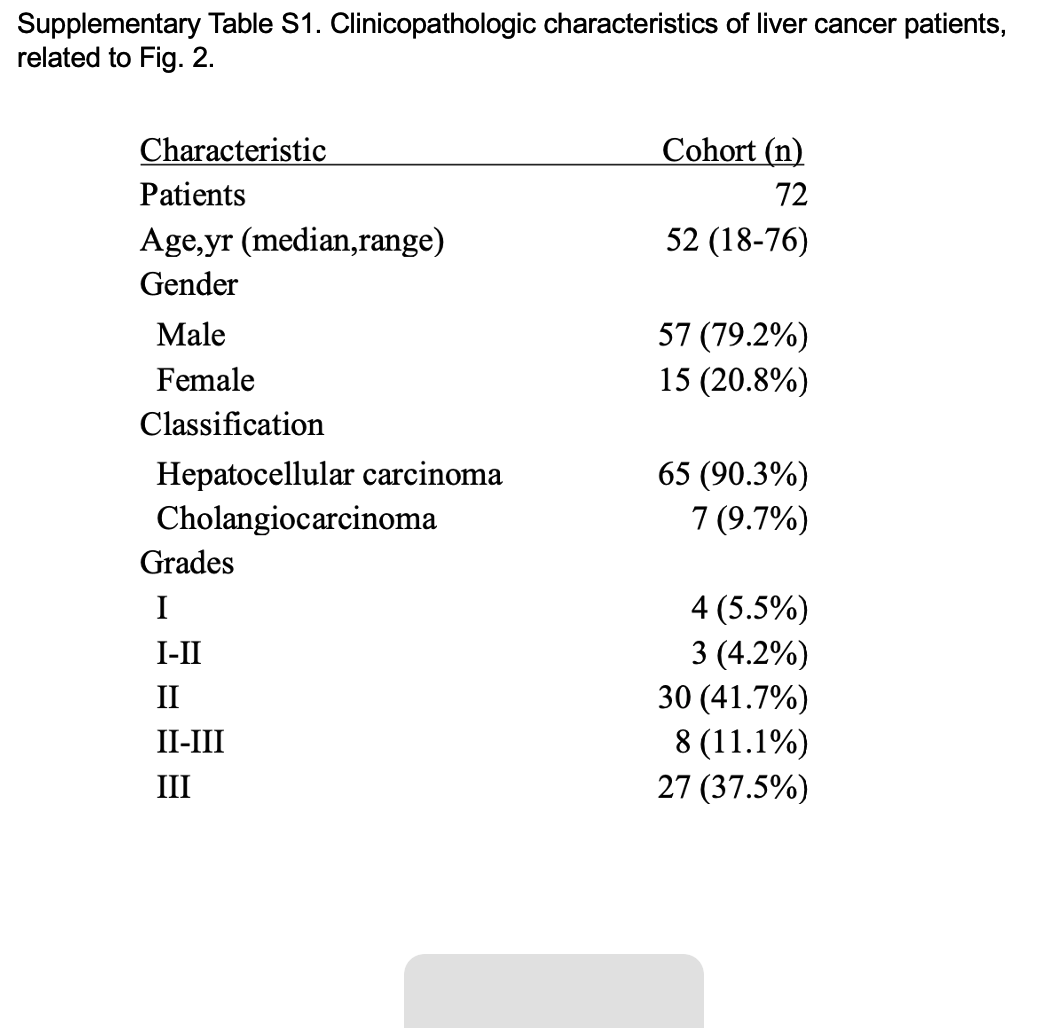
Table S1. Clinicopathologic characteristics of liver cancer patients, related to Fig. 2.
