# Supplementary figures and images for: Targeting PSAT1 to mitigate metastasis in tumors with p53-72Pro variant
Source: Signal Transduct Target Ther. 2023 Feb 15;8:65. doi: 10.1038/s41392-022-01266-7 (PMC9929071; doi:10.1038/s41392-022-01266-7)

Supplementary Fig. S1 PSAT1 is identified as a p53<sup>72P</sup>-interacting protein.

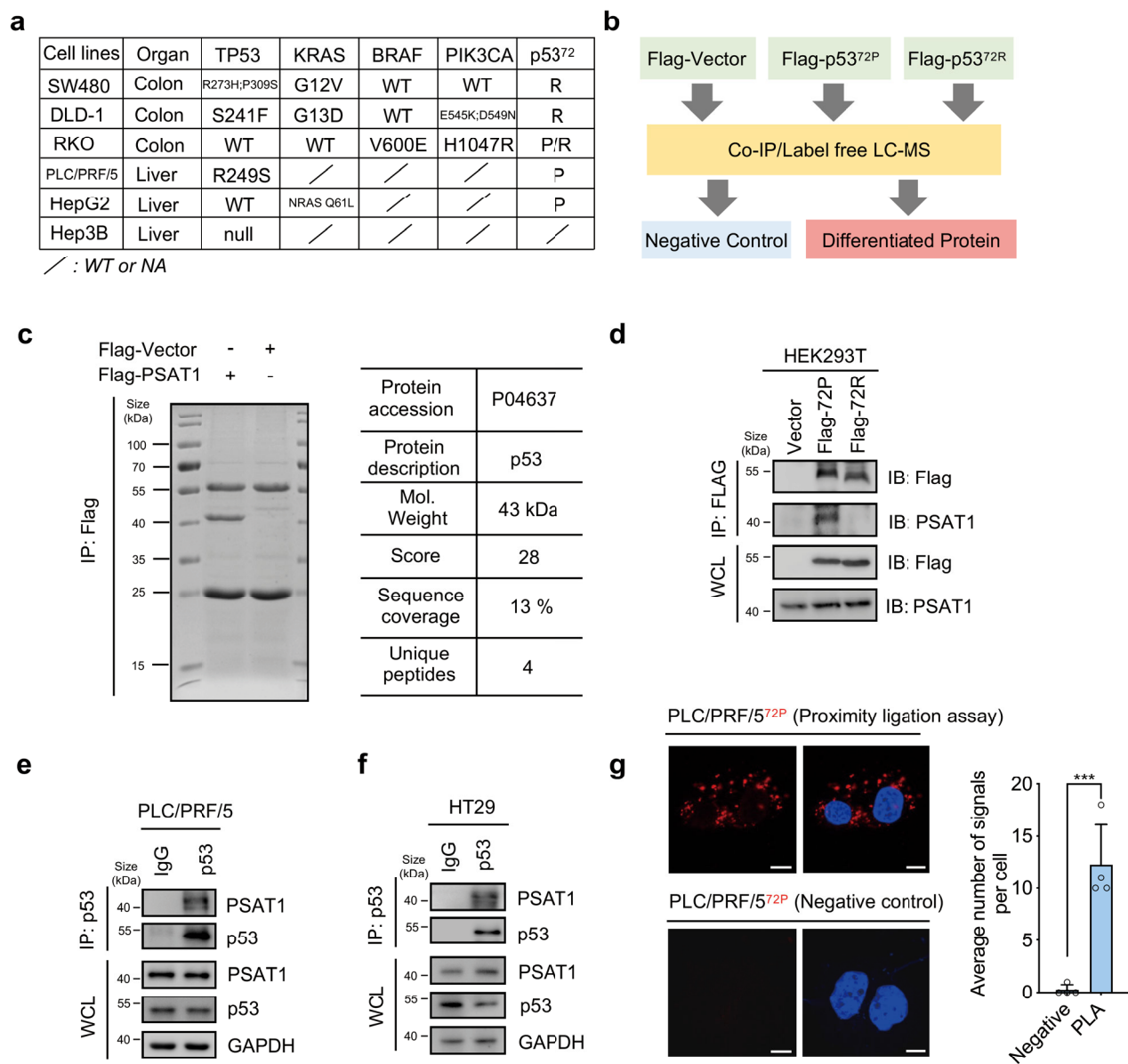

Supplement: Supplementary file 2 — Figure S1 [file 41392_2022_1266_MOESM2_ESM.pdf]

Supplementary Fig. S5 AOA impedes the interaction between PSAT1 and p53<sup>72P</sup>.

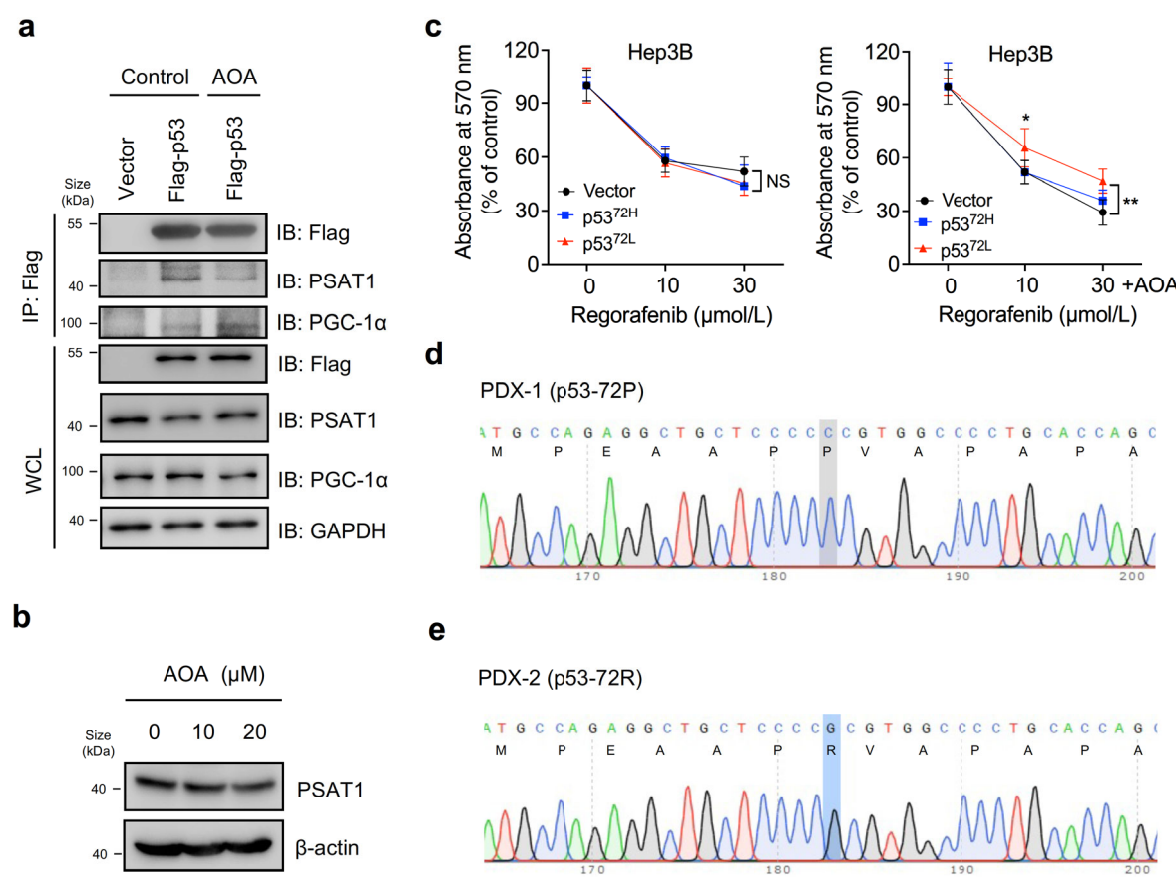

Supplement: Supplementary file 6 — Figure S5 [file 41392_2022_1266_MOESM6_ESM.pdf]
