# Supplementary material for: Targeting PSAT1 to mitigate metastasis in tumors with p53-72Pro variant
Source: Signal Transduct Target Ther. 2023 Feb 15;8:65. doi: 10.1038/s41392-022-01266-7 (PMC9929071; doi:10.1038/s41392-022-01266-7)

Supplementary Fig. S2 PSAT1 is essential for the metastatic potential of HCC cells containing p53[72P].

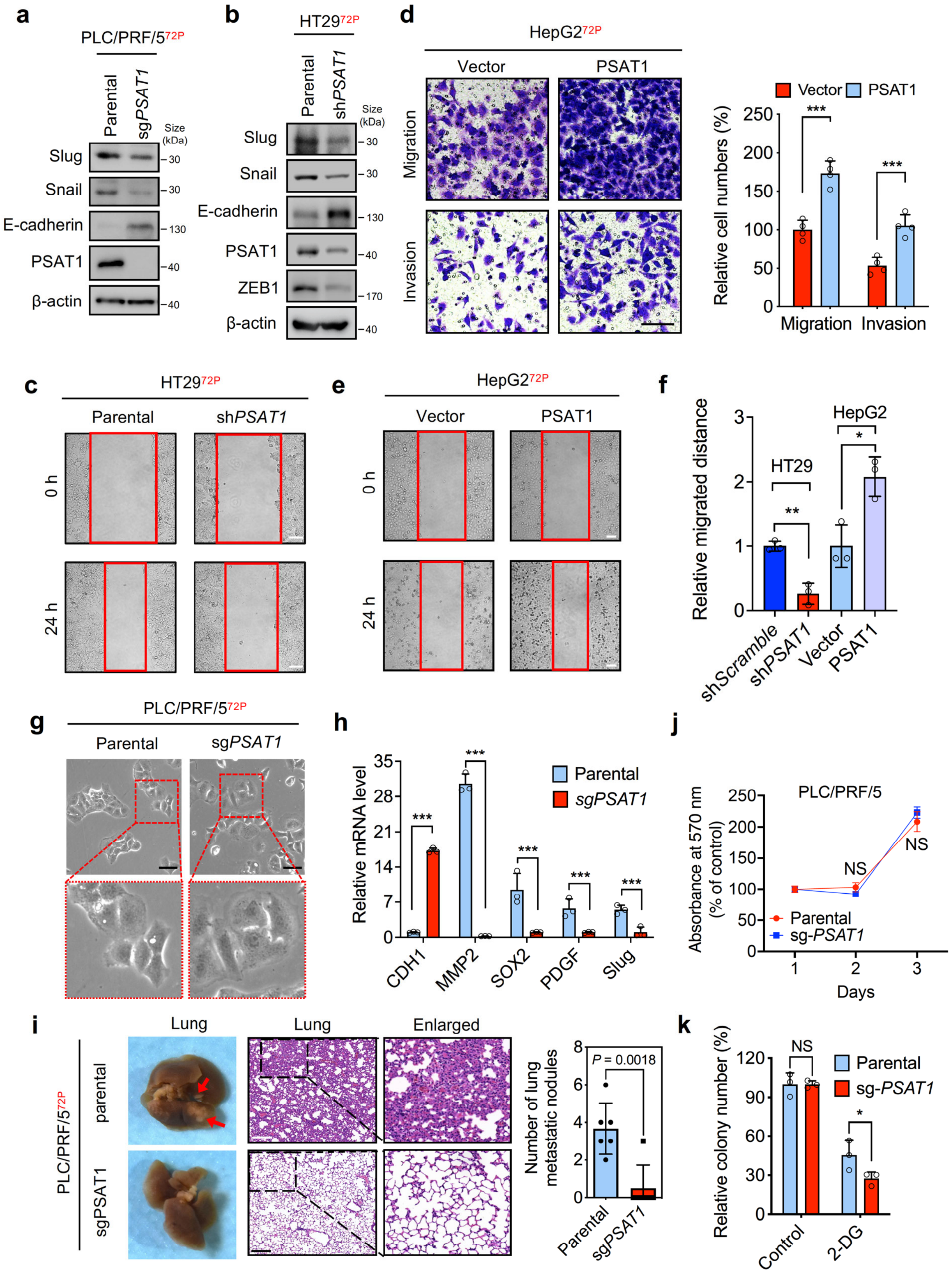

Supplement: Supplementary file 3 — Figure S2 [file 41392_2022_1266_MOESM3_ESM.pdf]
