# Supplementary material for: Targeting PSAT1 to mitigate metastasis in tumors with p53-72Pro variant
Source: Signal Transduct Target Ther. 2023 Feb 15;8:65. doi: 10.1038/s41392-022-01266-7 (PMC9929071; doi:10.1038/s41392-022-01266-7)

Supplementary Fig. S3 Loss of PSAT1 impedes mitochondria-related metabolic pathways in HCC cells.

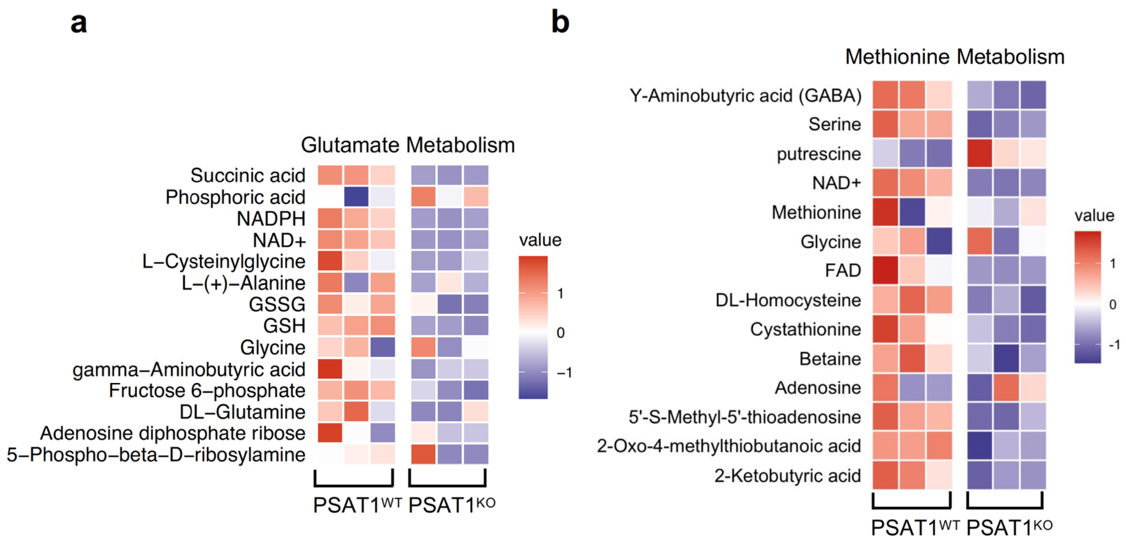

Supplement: Supplementary file 4 — Figure S3 [file 41392_2022_1266_MOESM4_ESM.pdf]
