# Supplementary material for: Targeting PSAT1 to mitigate metastasis in tumors with p53-72Pro variant
Source: Signal Transduct Target Ther. 2023 Feb 15;8:65. doi: 10.1038/s41392-022-01266-7 (PMC9929071; doi:10.1038/s41392-022-01266-7)
Supplement: Supplementary file 7 — Table S1 [file 41392_2022_1266_MOESM7_ESM.pdf]

Supplementary Table S1. Clinicopathologic characteristics of liver cancer patients, related to Fig. 2.

Characteristic	Cohort (n)
Patients	72
Age,yr (median,range)	52 (18-76)
Gender	
Male	57 (79.2%)
Female	15 (20.8%)
Classification	
Hepatocellular carcinoma	65 (90.3%)
Cholangiocarcinoma	7 (9.7%)
Grades	
I	4 (5.5%)
I-II	3 (4.2%)
II	30 (41.7%)
II-III	8 (11.1%)
III	27 (37.5%)
